# Photoinduced Fluorescence Changes in Selected Bacterial Strains Under 405 nm Laser Excitation: An In Vitro Hue-Based Monitoring Approach

**DOI:** 10.3390/mi17080885

**Published:** 2026-07-24

**Authors:** Agnieszka Urbańska, Magdalena Pajączkowska, Joanna Nowicka, Julia Kensy, Jan Kiryk, Łucja Janek, Rafał Wiench, Dariusz Skaba, Maciej Dobrzyński, Jacek Matys

**Affiliations:** 1 Department of Pediatric Dentistry and Preclinical Dentistry, Wroclaw Medical University, Krakowska 26, 50-425 Wroclaw, Poland; julia.kensy@student.umw.edu.pl (J.K.); maciej.dobrzynski@umw.edu.pl (M.D.); 2 Department of Microbiology, Faculty of Medicine, Wroclaw Medical University, Chalubinskiego 4, 50-368 Wroclaw, Poland; magdalena.pajaczkowska@umw.edu.pl (M.P.); joanna.nowicka@umw.edu.pl (J.N.); 3 Dental Surgery Department, Wroclaw Medical University, Krakowska 26, 50-425 Wroclaw, Poland; jan.kiryk@umw.edu.pl; 4 Statistical Analysis Centre, Wroclaw Medical University, Marcinkowskiego 2-6, 50-368 Wroclaw, Poland; lucja.janek@umw.edu.pl; 5 Department of Periodontal Diseases and Oral Mucosa Diseases, Faculty of Medical Sciences in Zabrze, Medical University of Silesia, 40-055 Katowice, Poland; rwiench@sum.edu.pl (R.W.); dskaba@sum.edu.pl (D.S.)

**Keywords:** autofluorescence, bacteria, 405 nm laser, image analysis, hue, ImageJ

## Abstract

Bacterial fluorescence generated by endogenous fluorophores may provide an optical approach for monitoring microbial fluorescence responses. The aim of this study was to analyse temporal changes in the Hue parameter, defined as an image-derived component of the HSB colour space representing the dominant colour tone, and to characterize strain-associated fluorescence response profiles under 405 nm violet-light excitation. A total of 28 reference bacterial strains were examined. Each strain was inoculated at eight distinct locations on a separate BHI agar plate, yielding eight colonies per strain. At 24, 48, 72, 96, and 168 h of incubation, the same colonies were repeatedly exposed for 45 s to a 405 nm continuous-wave diode laser operating at 200 mW and imaged in a completely darkened room using an iPhone 14, a yellow emission filter, and fixed acquisition geometry. The JPEG images were converted to the HSB colour space in ImageJ software (version 1.54), and background-corrected Hue values were calculated for each colony as ΔHue = Hmean − Hmean_BG using the corresponding colony and agar-background regions of interest. A complementary qualitative visual assessment was performed, and aligned rank transform ANOVA was used to evaluate the effects of bacterial strain, incubation time, and their interaction. ΔHue values differed significantly between strains and across incubation time points, with significant strain × time interactions observed in both rod-shaped and coccal bacteria (all *p* < 0.001). Exploratory planned contrasts also showed differences between the predefined Gram-staining groups (*p* = 0.0002) and between rod-shaped and coccal bacteria (*p* < 0.0001); however, these comparisons do not establish independent effects of Gram status or cellular morphology. The recorded fluorescence signal was generally weak, although visible temporal changes occurred in selected strains, including *Staphylococcus aureus* ATCC 6538P and *Proteus mirabilis* PCM 543. Overlap between ΔHue distributions limited separation of some strains. Background-corrected Hue analysis enabled exploratory within-dataset monitoring of temporal fluorescence colour changes under the applied in vitro imaging protocol. However, the overlap between ΔHue distributions and the technical limitations of the imaging system preclude the use of ΔHue as a standalone bacterial identification or diagnostic parameter.

## 1. Introduction

**Figure 1:**
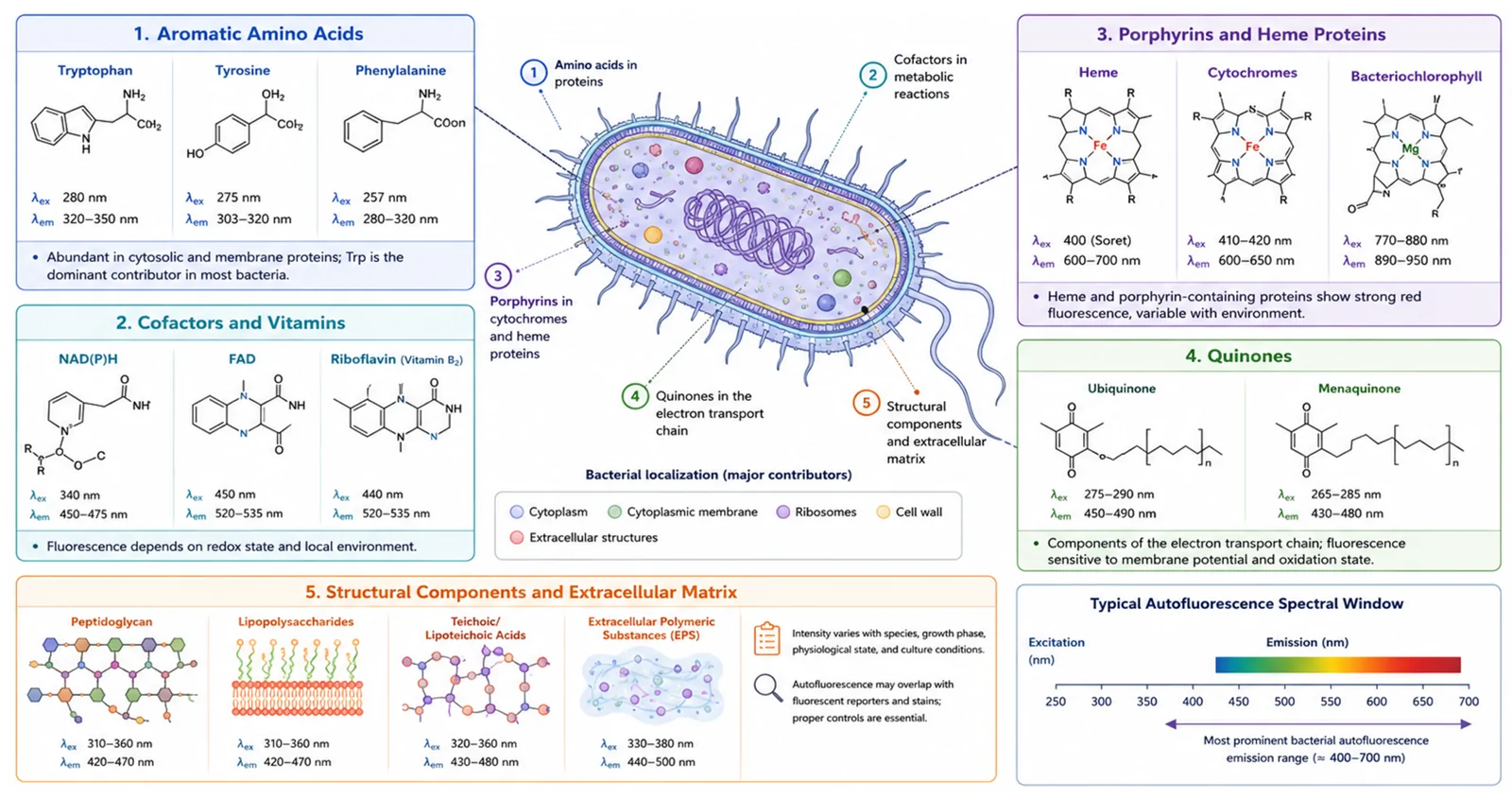
Bacterial sources of autofluorescence.

Autofluorescence is a natural phenomenon of light emission by living organisms after being excited by radiation of a specific wavelength, most commonly in the ultraviolet or violet range. In bacteria, this phenomenon results from the presence of endogenous fluorophores, such as molecules inherent to the cell, which, after absorbing energy, can emit light of a longer wavelength. The main endogenous bacterial fluorophores include coenzymes and redox-related molecules, such as NADH/NADPH and flavins, porphyrins and other intermediates of the haem biosynthesis pathway, and, in selected species such as *Pseudomonas aeruginosa*, the siderophore pyoverdine [1,2,3,4] (Figure 1). When a narrow-band 405 nm excitation source is used, these fluorophores are not excited equally [5,6,7,8,9]. Porphyrins are particularly relevant because their strong Soret-band absorption overlaps with the applied wavelength and their emission occurs predominantly in the red and red-orange spectral regions [10,11,12,13,14,15,16,17]. Flavins, including FAD and FMN, may contribute to the green-yellow component of the recorded signal, whereas pyoverdine may generate cyan or blue-green fluorescence in selected *P. aeruginosa* strains [18,19,20,21,22,23]. Although NADH and NADPH are important metabolic fluorophores, their contribution under exclusive 405 nm excitation should not be assumed to be dominant without spectroscopic confirmation [2,3,4].

The recorded optical response should therefore be interpreted as a composite emission arising from the overlapping contributions of several endogenous fluorophores whose relative abundance may vary with bacterial strain, metabolic activity, growth phase, nutrient availability, oxygen conditions, and physiological state [2,3,5,6,7,8,24,25,26]. Elastic scattering and reflection of the 405 nm excitation light are not fluorescence phenomena, because they do not involve emission at a longer wavelength. In the present system, a yellow emission filter was used to attenuate reflected and scattered excitation light. However, residual optical background and the possible formation or transformation of fluorescent photoinduced products cannot be completely excluded. Because no spectrally resolved measurements were performed, the observed Hue values cannot be assigned to a specific endogenous fluorophore, molecular process, or newly formed fluorescent species.

Bacterial fluorescence imaging, including approaches based on endogenous fluorophores, has also been explored as an optical method for visualizing microbial presence in clinical contexts, including wound assessment [27,28,29,30,31]. A similar rationale may apply in dentistry, where the oral cavity constitutes a complex ecological niche inhabited by numerous microbial species, and where the most common dental diseases, such as caries and periodontitis, are associated with the presence and maturation of biofilm [11,32,33,34,35,36]. Biofilm is a dynamic structure composed of multiple bacterial species that interact with one another and exhibit properties distinct from those of the individual cells or isolated species that comprise it [37,38,39,40]. For this reason, the ability to detect characteristic autofluorescence may represent a rapid, screening-based, and non-invasive approach for assessing the presence of mature biofilm or specific pathogens [32,33,41,42].

Previous research on bacterial autofluorescence has focused mainly on the presence of a fluorescent signal, its intensity, or spectral analysis [3,24,28,43]. However, signal intensity is susceptible to many technical factors, including the distance between the device and the object under examination, the angle of light incidence, exposure time, detector sensitivity, lighting conditions, and the background of the biological material [44,45,46,47]. For this reason, intensity alone may carry a substantial risk of error and limit the comparability of results between samples. Studies analysing the hue of autofluorescence itself, described as the ‘Hue’ parameter in the HSV/HSB colour space, are much less common. H, representing Hue, describes the colour tone, whereas S, representing Saturation, corresponds to colour intensity, and V/B, representing Value/Brightness, refers to image brightness [48,49]. In digital image analysis, Hue values may be stored not as percentages but as numerical values, for example on an 8-bit scale from 0 to 255 [50]. This conversion makes it possible to assign a numerical value to the observed colour and subject it to quantitative analysis [51].

The use of readily available imaging tools, including smartphones, may further enhance the practical potential of this approach [52,53,54]. It has been demonstrated that simple smartphone-based fluorescence imaging systems can be used for detection and documentation of bacterial signals under laboratory conditions [55,56,57]. This approach forms part of a broader trend toward the development of optical methods for bacterial detection that are rapid, non-contact, do not require amplification of genetic material, and can potentially be applied with minimal sample preparation [3,58,59,60,61]. The combination of accessible imaging platforms with quantitative image analysis may therefore provide new opportunities for standardized monitoring and characterization of bacterial fluorescence responses under controlled conditions [62,63,64,65,66,67,68,69].

The aim of this study was to quantify temporal changes in the image-derived Hue parameter in selected bacterial colonies exposed to 405 nm violet light and to determine whether the recorded temporal fluorescence response profiles differed among the analysed strains. Comparisons between the predefined Gram-staining and morphological groups were treated as exploratory analyses. The study was designed as an in vitro monitoring approach and was not intended to validate Hue as a standalone diagnostic or bacterial identification marker.

## 2. Materials and Methods

### 2.1. Bacterial Strains

A total of 28 reference bacterial strains were included in the study, comprising 14 rod-shaped and 14 coccal strains. The strains originated from the American Type Culture Collection (ATCC) (Manassas, VA, USA) and the Polish Collection of Microorganisms (PCM) (Wrocław, Poland). The rod-shaped group included representatives of the genera *Acinetobacter*, *Bacillus*, *Enterobacter*, *Escherichia*, *Klebsiella*, *Lactobacillus*, *Proteus*, *Pseudomonas*, and *Stenotrophomonas*, and comprised both Gram-positive and Gram-negative bacteria. The coccal group included Gram-positive bacteria belonging to the genera *Enterococcus*, *Staphylococcus*, and *Streptococcus*. In the statistical analysis, all strains were identified using abbreviated strain codes. Only reference bacterial strains were used in this in vitro study. No clinical specimens, biological fluids, nanoparticles, or colloidal systems were included. Detailed information on the analysed strains, including species names, collection numbers, strain codes, and Gram-staining characteristics, is presented in Table 1 and Table 2.

### 2.2. Bacterial Culture Conditions and Sample Preparation

The analysed strains were stored in a −80 °C freezer in tryptic soy broth (TSB; Biomaxima, Lublin, Poland) with the addition of 15% glycerol. Prior to each experimental cycle, the strains were cultured on TSA agar (Biomaxima, Lublin, Poland) at 37 °C under aerobic conditions for 24–48 h. A suspension with a density of 0.5 on the McFarland scale was prepared using a DEN-1 densitometer (Grant Instruments, Argenta, Poland) from fresh cultures of each bacterial strain. Next, a 5 µL aliquot of each standardized bacterial suspension was inoculated at eight distinct locations on a separate BHI agar plate (Biomaxima, Lublin, Poland), resulting in eight colonies per strain. The same plate and the same eight colonies were repeatedly exposed to 405 nm light and imaged after 24, 48, 72, 96, and 168 h of incubation.

### 2.3. Laser-Induced Fluorescence Imaging Procedure

Fluorescence excitation was performed using a 405 nm continuous-wave diode laser (SmartM, Lasotronix, Piaseczno, Poland). The output power was set to 200 mW, as indicated on the device display, and the laser system was calibrated before the experimental procedures. The entire agar plate containing eight colonies of the analysed strain was exposed to 405 nm violet light for 45 s. The handpiece was positioned approximately perpendicular to the agar surface at a fixed working distance of 11 cm from the sample. At the sample surface, the laser beam produced a circular illumination spot with a diameter of approximately 8 mm, corresponding to a radius of 4 mm, or 0.4 cm. The illuminated area was calculated using the formula: A = πr^2^ = π × (0.4 cm)^2^ = 0.503 cm^2^

Based on the laser output power of 200 mW, the irradiance at the sample surface was calculated as: Irradiance = Power/Area = 200 mW/0.503 cm^2^ = 397.6 mW/cm^2^

Thus, the irradiance was approximately 398 mW/cm^2^, rounded to 400 mW/cm^2^. For the 45 s exposure time, the delivered radiant exposure was calculated as: Radiant exposure = Irradiance × time = 0.398 W/cm^2^ × 45 s = 17.9 J/cm^2^

This configuration enabled consistent illumination of individual bacterial colonies under standardized experimental conditions. The smartphone camera served as the direct fluorescence-image detector; no additional scientific camera, photodetector, or intermediate imaging system was used. Immediately after laser excitation, fluorescence images were recorded during laser irradiation using a smartphone camera (iPhone 14, Apple, Cupertino, CA, USA) positioned 19 cm from the sample. A yellow emission filter was used to reduce reflected excitation light and allow transmission of longer wavelengths associated with photoinduced fluorescence emission in the green-to-red spectral range. This enabled clear visualization of fluorescence signals that would otherwise have been masked by the violet excitation light. All images were acquired in a completely darkened room to eliminate uncontrolled ambient illumination. The 405 nm laser was the only excitation-light source used during image acquisition. The same smartphone, yellow emission filter, laser parameters, camera-to-sample distance, and acquisition geometry were maintained throughout the experiment. The images were saved in JPEG format. The white balance and the standard exposure programme were active, while no flash was used. No manual post-acquisition colour enhancement was applied. However, proprietary device-level image processing, including automatic white-balance adjustment, tone mapping, and computational image enhancement, could not be completely disabled or independently controlled. Images were not acquired in RAW/DNG format, and no physical colour-calibration target or calibrated fluorescence reference standard was used.

### 2.4. Digital Image Analysis and Region-of-Interest Selection

The acquired RGB images were imported into ImageJ software (version 1.54) for further processing. Each image was analysed separately to ensure consistency. The images were transformed from the RGB colour model to the HSB colour space using the HSB Stack function, and the Hue component was isolated for quantitative evaluation. Hue values were expressed on a scale ranging from 0 to 255. For each agar plate, regions of interest (ROIs) were manually designated. These included eight colony ROIs corresponding to the same eight colonies repeatedly followed on each strain-specific agar plate (c1–c8), together with one background ROI selected on a colony-free area of the same plate. Background ROIs were consistently selected on the agar surface to reduce potential bias related to uneven illumination. Colony ROIs were defined as circular regions, carefully avoiding colony edges in order to minimize the influence of optical artifacts and local illumination variations. Within each ROI, the minimum (Hmin), mean (Hmean), and maximum (Hmax) Hue values were determined. In addition, the mean Hue value of the background area (Hmean_BG) was calculated for each plate. The primary parameter used for statistical analysis was the background-corrected Hue value, calculated for each colony as ΔHue = Hmean − Hmean_BG, where Hmean represents the mean Hue value of the colony ROI and Hmean_BG represents the mean Hue value of the agar-background ROI obtained from the corresponding plate. Because the observed Hue values did not cross the 0/255 boundary, direct subtraction was appropriate and circular correction was not required.

### 2.5. Visual Characterization of Autofluorescence in Bacterial Colonies

Alongside the quantitative processing of images, a descriptive visual evaluation of fluorescence was performed for all incubation time points. This step aimed to confirm the presence of autofluorescence in the examined bacterial colonies and to document visible changes occurring during their growth. The assessment was carried out by a single trained observer familiar with fluorescence-based evaluation of microbial samples. Several visual characteristics were taken into account, including whether a fluorescence signal could be detected, its perceived strength, colour appearance, and how it was distributed within the colony. Additionally, temporal changes in these features were analysed by comparing images obtained at successive incubation stages. Special attention was given to the pattern of signal distribution, distinguishing between evenly distributed fluorescence across the colony surface and signals concentrated in specific localized regions. This qualitative approach was used solely to complement the numerical analysis based on hue values and did not serve as a basis for statistical grouping or classification. The qualitative assessment was designed as a complementary visual evaluation based on predefined image characteristics rather than as a measurement of absorbance or absolute fluorescence intensity. For each strain and incubation time point, the acquired images were assessed for: (1) the presence or absence of a visually detectable fluorescence signal; (2) perceived signal intensity; (3) colour appearance; (4) spatial distribution of the signal within the colony; and (5) temporal changes in these characteristics across successive incubation stages. The same standardized images used for the quantitative Hue analysis were evaluated by a trained observer. The qualitative assessment was descriptive and complementary and was not used for statistical classification, statistical grouping, or bacterial identification. All inferential statistical analyses were based exclusively on the background-corrected ΔHue values.

### 2.6. Statistical Analysis

Data were analysed in R (version 4.5.3). The primary dependent variable was the background-corrected Hue value, calculated for each colony as ΔHue = Hmean − Hmean_BG, where Hmean represented the mean Hue value of the colony region of interest and Hmean_BG represented the mean Hue value of the corresponding agar-background region. Because the observed Hue values did not cross the 0/255 boundary, direct subtraction was appropriate and no circular correction was required. Before inferential analysis, normality and homogeneity of variance were assessed using the Shapiro–Wilk and Levene tests, respectively. Potential outliers were evaluated using boxplots based on the 1.5 × IQR criterion. No observations were excluded from the analysis. Because the assumptions required for parametric factorial ANOVA were not fulfilled, a rank-based aligned rank transform analysis of variance (ART-ANOVA) was applied. Four predefined hypotheses were tested. H1 assumed that ΔHue values differ significantly between bacterial strains. H2 assumed that ΔHue values change significantly across incubation time points. H3 assumed that temporal ΔHue profiles differ between strains, corresponding to a significant strain × time interaction. H4 assumed that ΔHue values differ between the predefined Gram-staining groups and between rod-shaped and coccal bacteria. H1–H3 were evaluated separately for rod-shaped and coccal bacteria using ART-ANOVA, with bacterial strain, incubation time, and their interaction included as factors. Following significant omnibus effects, post hoc pairwise comparisons were performed using ART contrasts with Holm adjustment for multiple testing. H4 was evaluated using predefined ART contrasts comparing Gram-positive with Gram-negative bacteria and rod-shaped with coccal bacteria. These group-level contrasts were treated as exploratory because Gram status and cellular morphology were not fully independent in the selected strain panel. The significance threshold was set at *p* < 0.05.

## 3. Results

The results are presented in a sequential manner. First, the effects of bacterial strain, incubation time, and their interaction on background-corrected ΔHue values are reported. Exploratory comparisons between the predefined Gram-staining and morphological groups are then presented, followed by strain-specific temporal profiles, an assessment of the overlap between ΔHue distributions, and a complementary qualitative assessment of the recorded fluorescence signal illustrated in Section 3.5.

### 3.1. Effect of Bacterial Strain and Incubation Time on Background-Corrected Hue Values (ΔHue)

The quantitative background-corrected Hue analysis showed that both bacterial strain and incubation time significantly influenced ΔHue values (Hmean − Hmean_BG) under 405 nm laser excitation. Because the assumptions for parametric ANOVA were not met, the data were analysed using aligned rank transform ANOVA. In rod-shaped bacteria, ART-ANOVA revealed significant effects of strain (F(13,490) = 398.22; *p* < 0.001) and incubation time (F(4490) = 419.95; *p* < 0.001) on ΔHue values, indicating differences between strains and changes across incubation time points after correction for the agar background. The strain × time interaction was also significant (F(52,490) = 44.74; *p* < 0.001), showing that the temporal pattern of ΔHue changes depended on the strain. These results support H1, H2, and H3 for rod-shaped bacteria and justify rejection of the corresponding null hypotheses (Table 3).

In coccal bacteria, ART-ANOVA also showed significant effects of bacterial strain (F(13,490) = 323.712; *p* < 0.001), incubation time (F(4490) = 85.452; *p* < 0.001), and the strain × time interaction (F(52,490) = 25.375; *p* < 0.001). This indicates that ΔHue values differed between coccal strains, changed significantly across incubation time points, and followed strain-specific temporal trajectories after correction for the agar background. These findings also support H1, H2, and H3 for coccal bacteria. Accordingly, the corresponding null hypotheses were rejected (Table 4).

The results show that the background-corrected image-derived colour parameter, ΔHue, is influenced by both bacterial strain and incubation time. The significant strain × time interactions indicate that the temporal dynamics of ΔHue changes cannot be generalized across all strains and should be interpreted in a strain-specific manner. Because all principal effects remained significant after subtraction of the corresponding agar-background Hue value, the main strain- and time-related findings were robust to background correction. Exploratory comparisons between the predefined broader bacterial groups were therefore performed next.

### 3.2. Differences Between Gram-Positive and Gram-Negative Bacteria and Between Rod-Shaped and Coccal Bacteria in Background-Corrected Hue Values

To examine whether background-corrected Hue values differed between the predefined broader bacterial groups, planned ART contrasts were performed. The analysis demonstrated significant differences between Gram-positive and Gram-negative bacteria, as well as between rod-shaped and coccal bacteria. These contrasts were based on ΔHue values (Hmean − Hmean_BG), confirming that the group-level differences remained significant after correction for the agar background. However, because Gram status and cellular morphology were not fully independent in the selected strain panel, these contrasts should be interpreted as exploratory group-level comparisons rather than evidence of independent effects of Gram status or cell shape.

The planned contrasts showed significant differences between Gram-positive and Gram-negative bacteria (estimate = 73.5; SE = 19.4; df = 1110; t = 3.788; *p* = 0.0002) and between rod-shaped and coccal bacteria (estimate = −86.3; SE = 19.2; df = 1110; t = −4.503; *p* < 0.0001). Within the composition of the present strain panel, the predefined Gram-positive group showed higher ΔHue values than the predefined Gram-negative group. This descriptive group-level difference cannot be interpreted as an independent effect of Gram status because Gram staining, morphology, strain identity, and plate identity were not separable in the present design. However, because Gram status and cellular morphology were confounded in the selected strain panel, these findings do not establish independent discriminatory effects of either variable (Table 5). Because these group-level contrasts do not describe the heterogeneous temporal responses of individual strains, the subsequent analysis focused on strain-specific ΔHue profiles across the incubation period.

### 3.3. Strain-Specific Temporal Patterns of Background-Corrected Hue Changes

**Figure 2:**
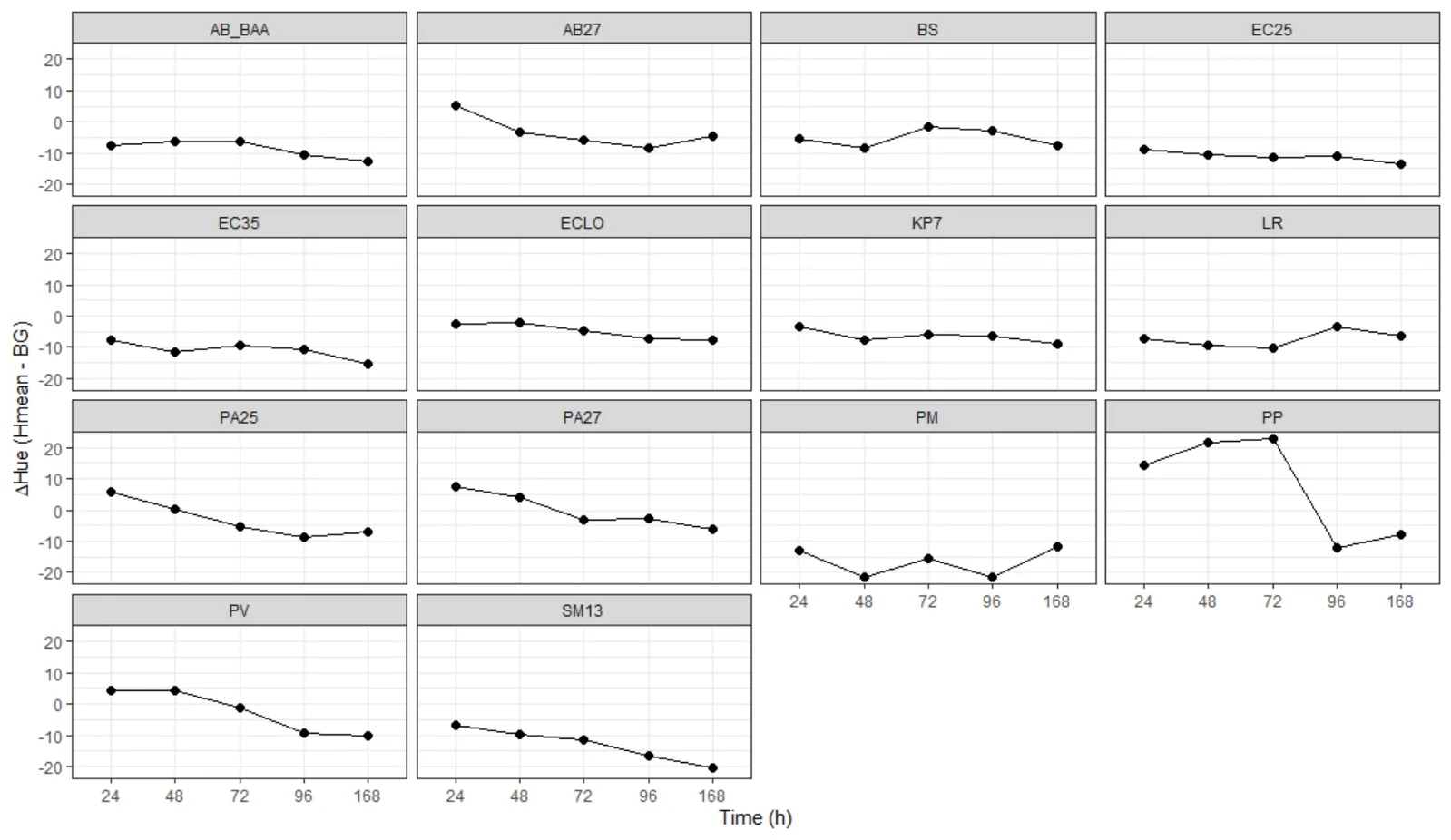
Temporal changes in background-corrected mean Hue values (ΔHue = Hmean − Hmean_BG) for rod-shaped bacterial strains during incubation. The plotted points represent mean ΔHue values. The corresponding within-strain distributions and variability at each incubation time point are presented in Figure 3.

Following the significant strain × time interactions, temporal ΔHue profiles were examined separately for individual rod-shaped and coccal strains to characterize the direction and variability of the background-corrected colour changes.

n rod-shaped bacteria, several strains showed an overall decrease in ΔHue values during incubation. A progressive decrease was observed for *Stenotrophomonas maltophilia* ATCC 13637, whereas *Pseudomonas aeruginosa* ATCC 2562 showed a decrease up to 96 h followed by a slight increase at 168 h. Other strains displayed more stable or non-linear temporal profiles. For example, *Proteus mirabilis* PCM 543 showed pronounced non-linear fluctuations, whereas *Lactobacillus rhamnosus* ATCC 9595 remained relatively stable during the early incubation period and exhibited a transient increase at 96 h. These differences indicate that the dynamics of background-corrected fluorescence colour changes were not uniform among rod-shaped bacterial strains (Figure 2).

**Figure 3:**
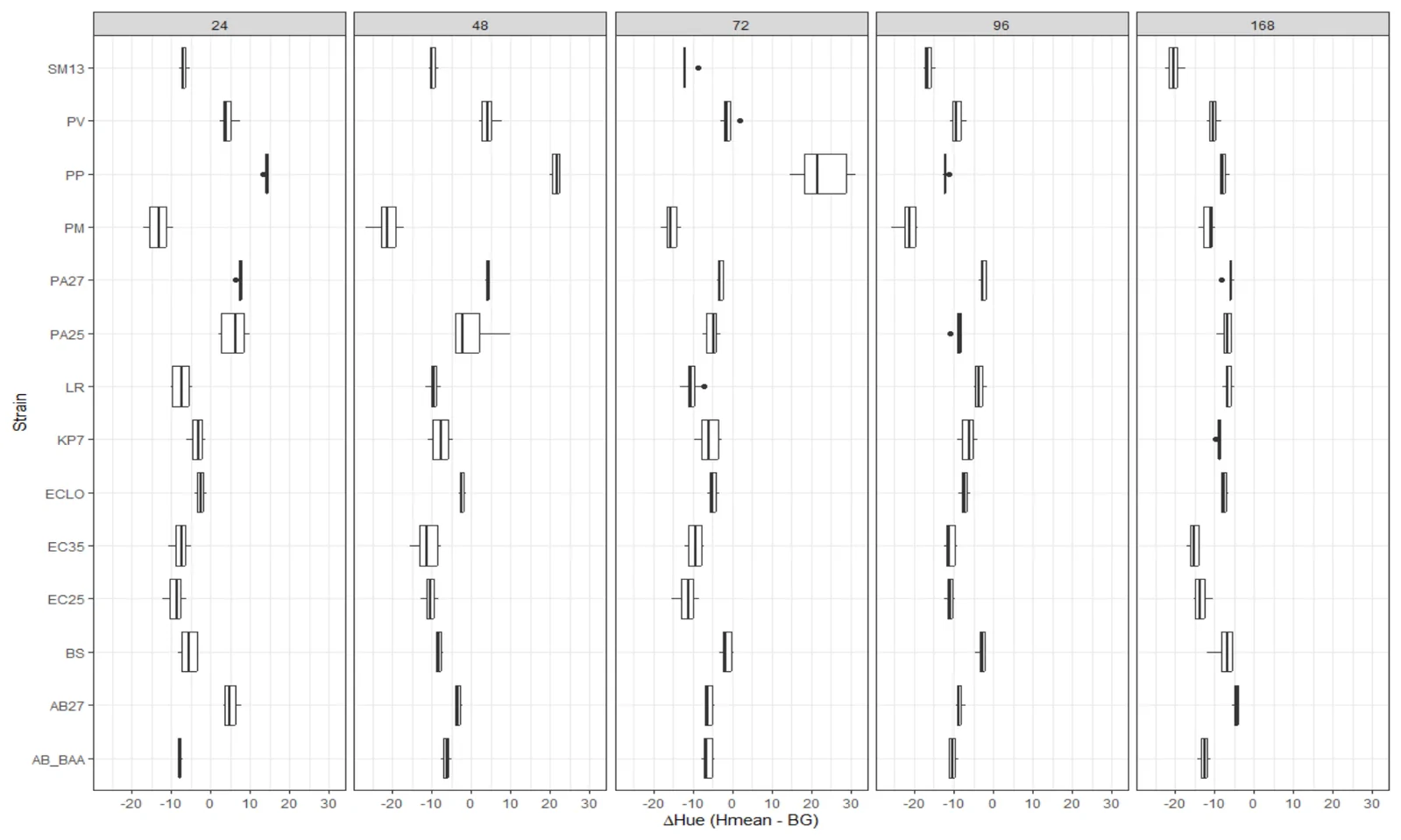
Distribution of background-corrected Hue values (ΔHue = Hmean − Hmean_BG) for rod-shaped bacterial strains across incubation time points. Dots represent outlier values.

The distribution of ΔHue values across incubation time points showed clear differences between rod-shaped bacterial strains in terms of median values and value ranges. *Pseudomonas aeruginosa* ATCC 2562 and *Stenotrophomonas maltophilia* ATCC 13637 showed an overall decrease in ΔHue, whereas *Proteus mirabilis* PCM 543 and *Lactobacillus rhamnosus* ATCC 9595 exhibited more pronounced non-linear changes. A distinct transient increase and wider distribution of ΔHue values at 72 h were also observed for *Pseudomonas putida* ATCC 33015/PCM 2152. Despite partial overlap between several strains, shifts in median values and changes in data dispersion across incubation time points were evident. These findings confirm that the temporal distribution of background-corrected Hue values differed between rod-shaped bacterial strains (Figure 3).

**Figure 4:**
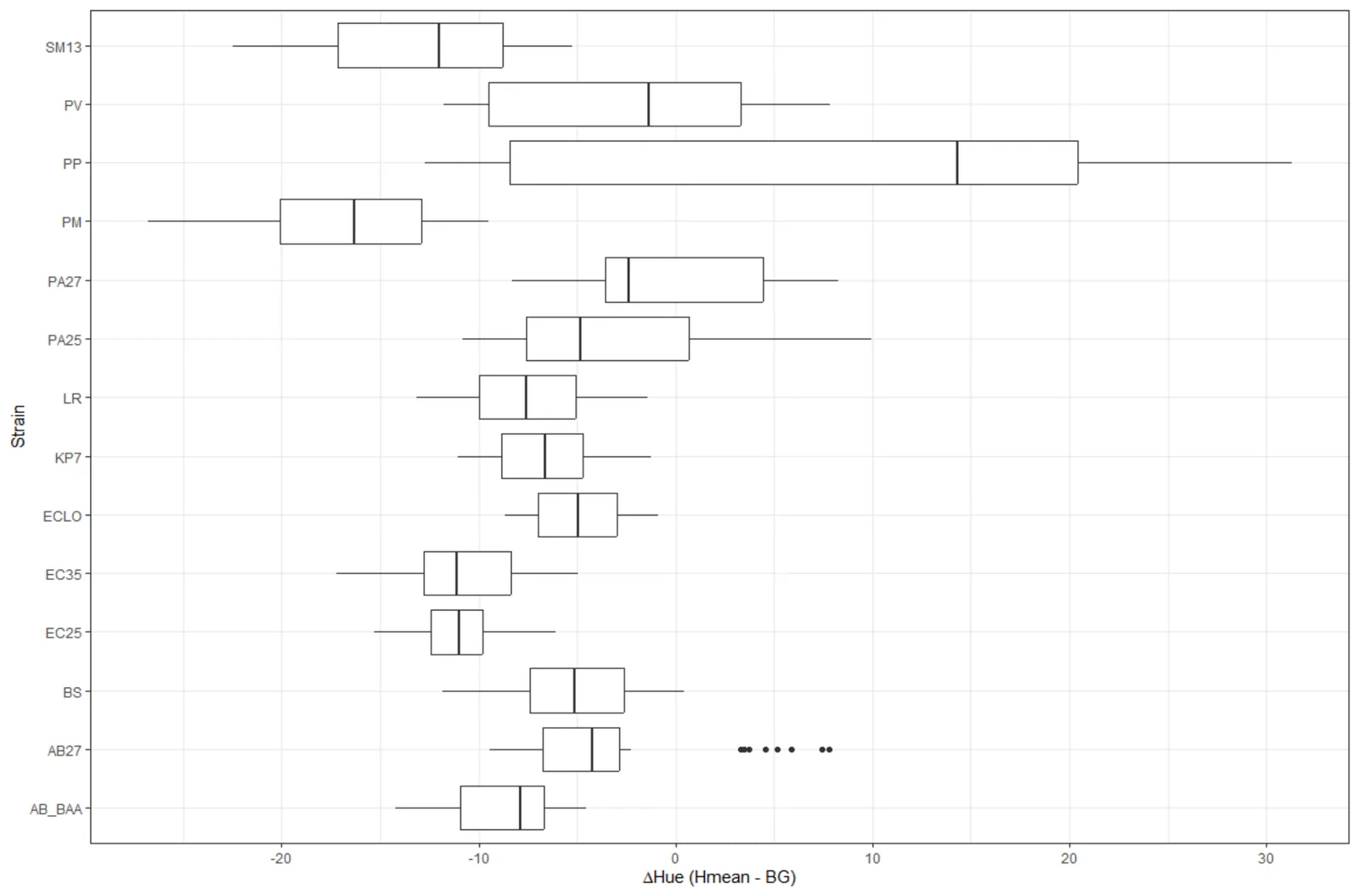
Overall distribution of background-corrected Hue values (ΔHue = Hmean − Hmean_BG) among rod-shaped bacterial strains. Dots represent outlier values.

The distribution of ΔHue values among rod-shaped bacterial strains showed clear differences in median values and variability. Relatively high and broadly distributed ΔHue values were observed for *Pseudomonas putida* ATCC 33015/PCM 2152, whereas lower median values were recorded for *Proteus mirabilis* PCM 543 and *Stenotrophomonas maltophilia* ATCC 13637. *Pseudomonas vulgaris* PCM 2269 also showed a relatively broad distribution, while several strains, including *Klebsiella pneumoniae* ATCC 700603 and *Enterobacter cloacae* PCM 2569, exhibited narrower distributions. However, partial overlap in ΔHue ranges was observed between several strains, particularly in the intermediate range, indicating that ΔHue alone may have limited discriminatory power for some rod-shaped bacteria. Because values from all incubation time points were combined in this analysis, the observed distribution widths reflect both temporal variation and variability among replicates (Figure 4).

**Figure 5:**
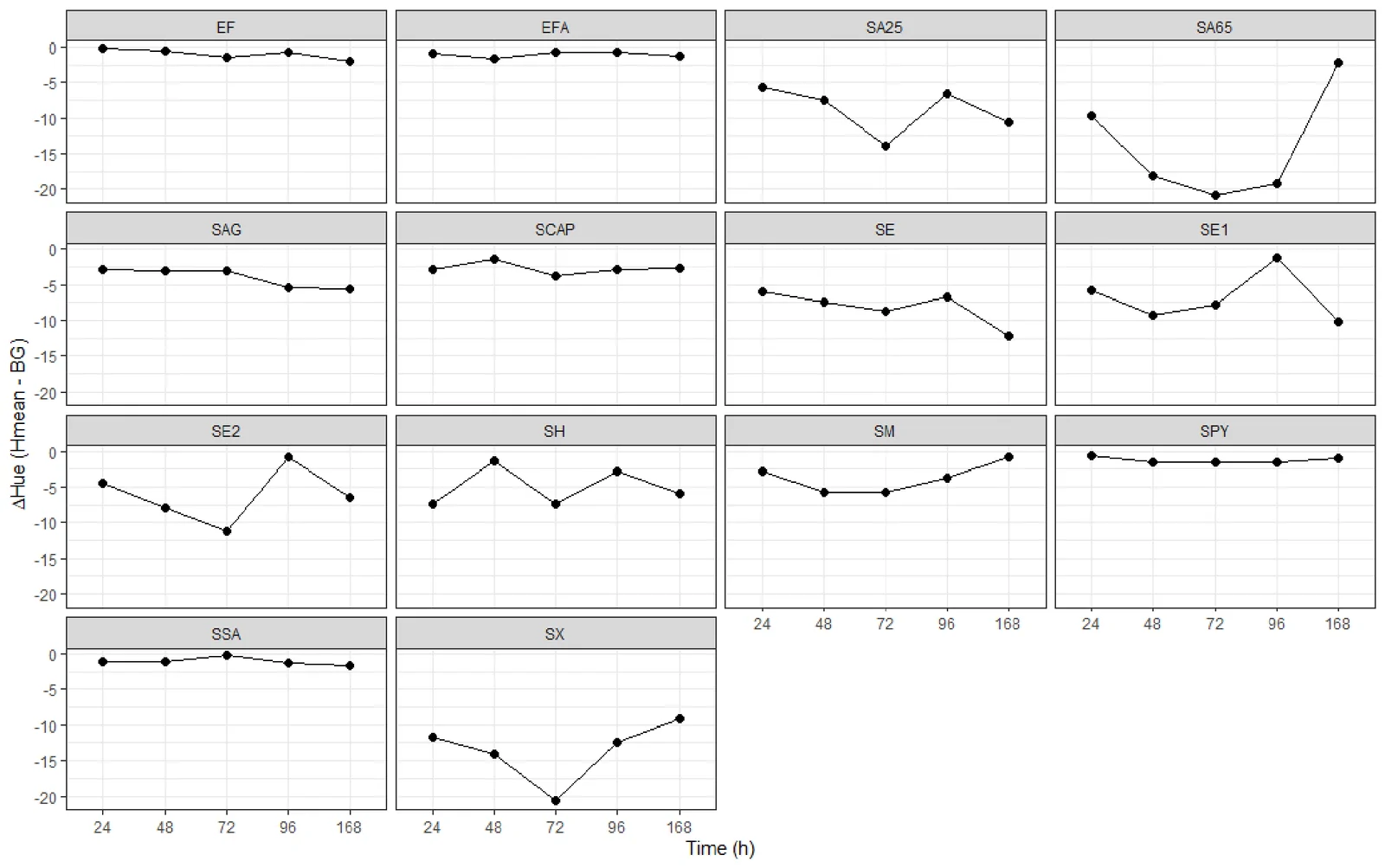
Temporal changes in background-corrected mean Hue values (ΔHue = Hmean − Hmean_BG) for coccal bacterial strains during incubation. The plotted points represent mean ΔHue values. The corresponding within-strain distributions and variability at each incubation time point are presented in Figure 6.

In coccal bacteria, strain-specific temporal profiles were also observed. Relatively stable ΔHue values were recorded for *Enterococcus faecalis* ATCC 29212, *Enterococcus faecium* PCM 1859, *Streptococcus pyogenes* ATCC 12344, and *Streptococcus sanguinis* ATCC 10556. In contrast, *Staphylococcus aureus* ATCC 6538P and *Staphylococcus xylosus* ATCC 700404 showed pronounced non-linear profiles, with decreases up to 72–96 h followed by increases at 168 h. Non-linear changes were also observed for *Staphylococcus aureus* ATCC 25923, *Staphylococcus epidermidis* ATCC 2479 and ATCC 2532, and *Staphylococcus haemolyticus* ATCC 2113. *Streptococcus mutans* ATCC 25175 showed a gradual increase in ΔHue at the later incubation time points. The direction and magnitude of the changes differed between strains, supporting the strain-dependent character of the background-corrected fluorescence response (Figure 5).

**Figure 6:**
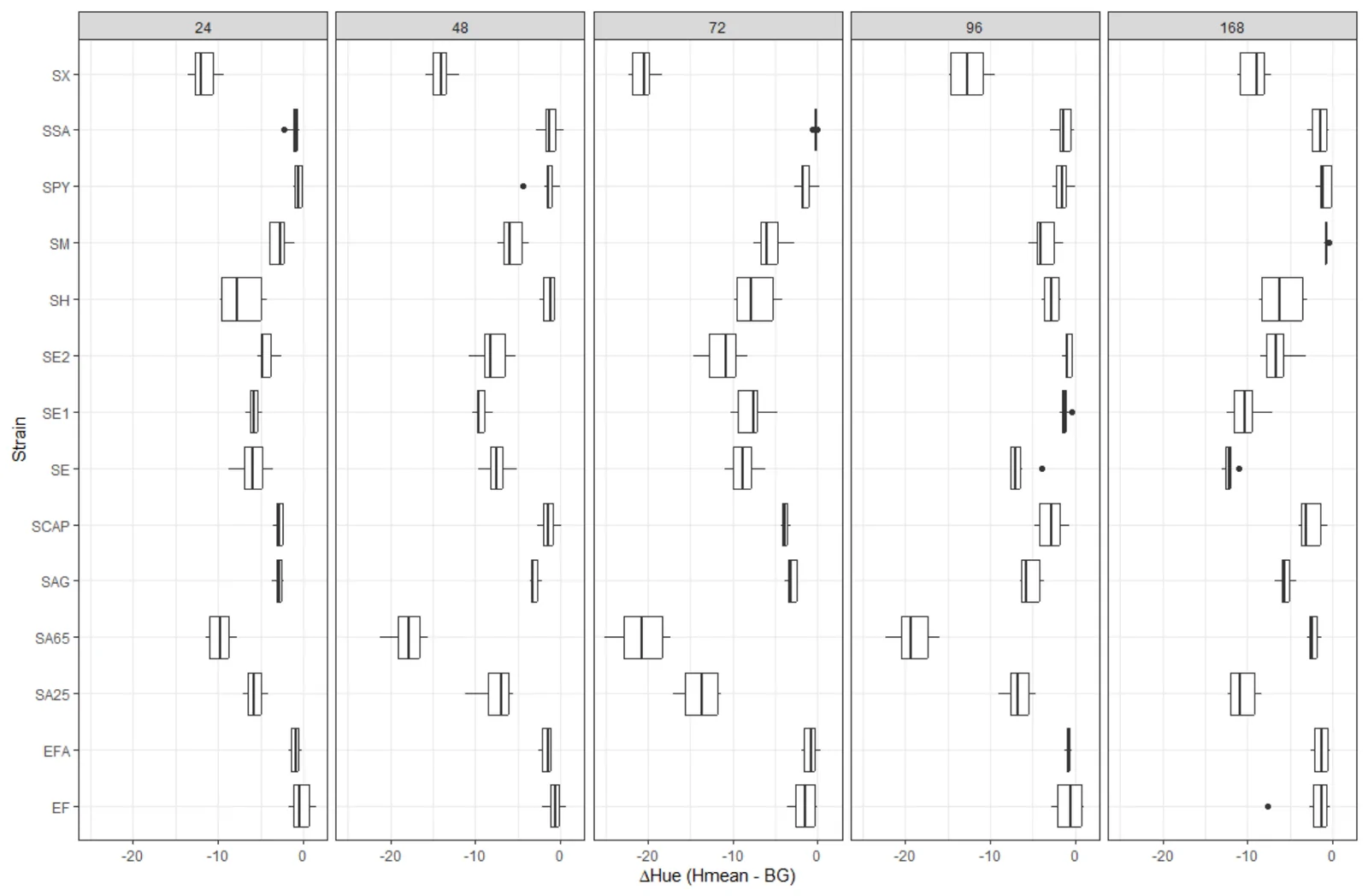
Distribution of background-corrected Hue values (ΔHue = Hmean − Hmean_BG) in coccal bacterial strains across incubation time points. Dots represent outlier values.

The distribution of ΔHue values in coccal bacteria across incubation time points confirmed that both strain identity and incubation time influenced the analysed parameter. Differences in median values and data dispersion were observed between strains at each time point, and the relative position of several strains changed during incubation. For example, *Staphylococcus aureus* ATCC 6538P showed progressively lower ΔHue values between 24 and 72 h, followed by a marked increase at 168 h. *Staphylococcus xylosus* ATCC 700404 exhibited a gradual shift towards higher, less negative ΔHue values at the later incubation stages. In contrast, *Enterococcus faecalis* ATCC 29212, *Enterococcus faecium* PCM 1859, and *Streptococcus pyogenes* ATCC 12344 maintained relatively stable ΔHue values throughout the incubation period. These observations further support the presence of a significant strain × time interaction after correction for the agar background (Figure 6).

**Figure 7:**
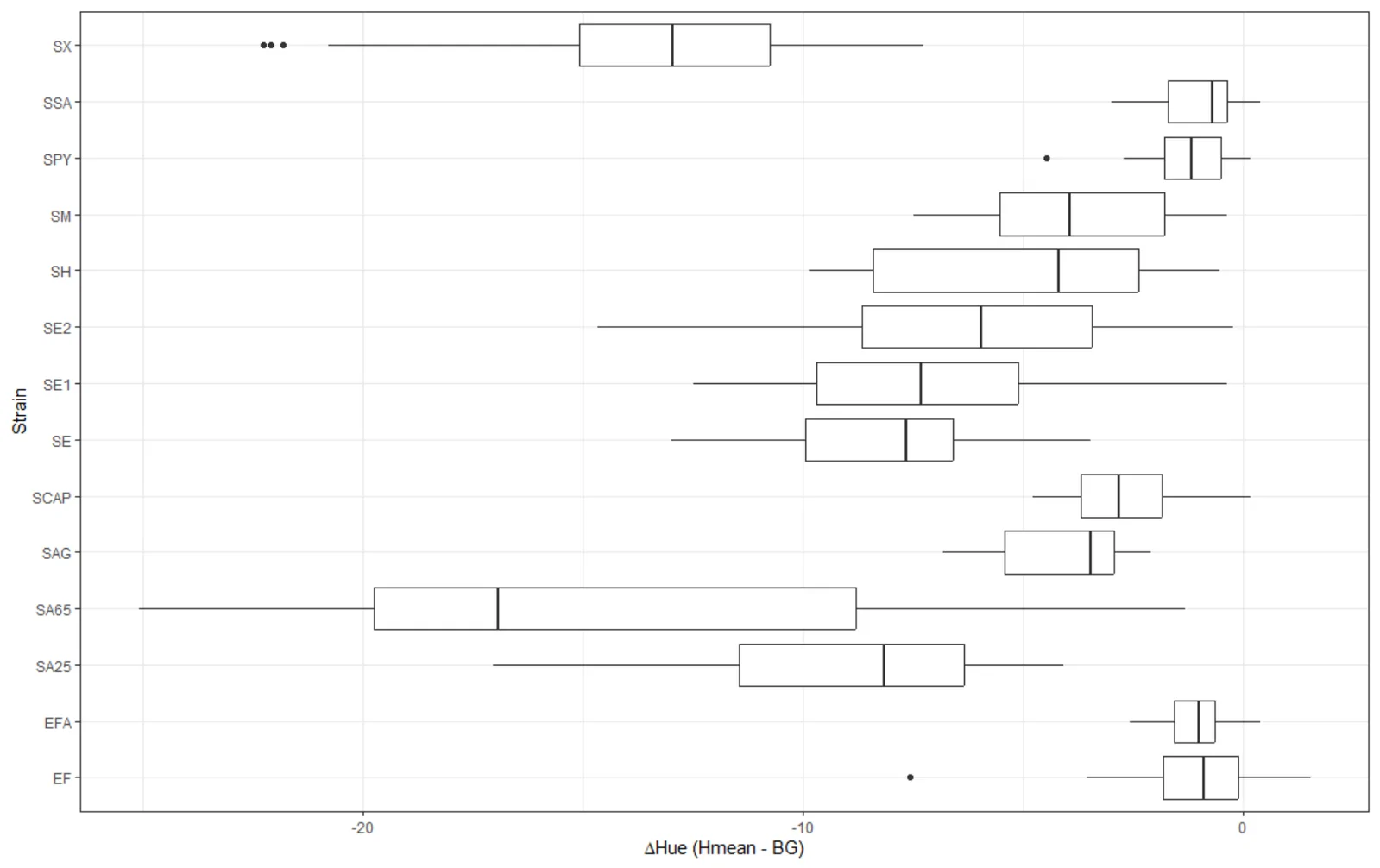
Overall distribution of background-corrected Hue values (ΔHue = Hmean − Hmean_BG) among coccal bacterial strains. Dots represent outlier values.

The distribution of ΔHue values among coccal bacterial strains showed clear differences between strains, although partial overlap was present. The highest, least negative ΔHue values were observed for *Enterococcus faecalis* ATCC 29212, *Enterococcus faecium* PCM 1859, *Streptococcus pyogenes* ATCC 12344, and *Streptococcus sanguinis* ATCC 10556. In contrast, the lowest median ΔHue values were recorded for *Staphylococcus aureus* ATCC 6538P and *Staphylococcus xylosus* ATCC 700404. Relatively broad distributions were observed particularly for *S. aureus* ATCC 6538P and *S. xylosus* ATCC 700404, as well as for *Staphylococcus haemolyticus* ATCC 2113 and *Streptococcus mutans* ATCC 25175, indicating greater within-strain variability in the pooled dataset. These findings support H1, although the observed overlap suggests that ΔHue alone may have limited discriminatory value for some coccal bacterial strains. Because data from all incubation time points were combined, the distribution widths reflect both temporal variation and variability among replicates (Figure 7).

Post hoc analyses using ART contrasts with Holm correction revealed numerous significant pairwise differences between bacterial strains at individual incubation time points for the background-corrected ΔHue parameter. The greatest differentiation between strains was generally observed after 72–168 h of incubation, although the number and pattern of significant comparisons varied according to incubation time. These observations further support H1, H2, and H3, confirming that ΔHue values differed between strains, changed across incubation time points, and followed strain-specific temporal profiles after correction for the agar background.

Among rod-shaped bacteria, relatively low ΔHue values were observed for *Proteus mirabilis* PCM 543 and *Stenotrophomonas maltophilia* ATCC 13637, whereas relatively high and more variable values were observed for *Pseudomonas putida* ATCC 33015/PCM 2152. *Pseudomonas aeruginosa* ATCC 27853 also showed comparatively higher ΔHue values than several other rod-shaped strains. In the coccal bacteria group, the highest, least negative ΔHue values were observed for *Enterococcus faecalis* ATCC 29212, *Enterococcus faecium* PCM 1859, *Streptococcus pyogenes* ATCC 12344, and *Streptococcus sanguinis* ATCC 10556, whereas lower and broader distributions were observed particularly for *Staphylococcus aureus* ATCC 6538P and *Staphylococcus xylosus* ATCC 700404.

These descriptive distribution patterns complement the post hoc findings but do not imply unequivocal separation between all strains, because substantial overlap remained. Detailed pairwise comparison results are presented in the Supplementary Materials, Tables S3–S11. Figures S1 and S2 present the boxplots used for the assessment of potential outliers.

### 3.4. Overall Overlap and Separation of Background-Corrected Hue Distributions

The distributions of background-corrected Hue values (ΔHue) differed between bacterial strains in both rod-shaped and coccal bacteria. In rod-shaped bacteria, some strains occupied relatively distinct regions of the ΔHue range. Lower ΔHue values were observed for *Proteus mirabilis* PCM 543 and *Stenotrophomonas maltophilia* ATCC 13637, whereas higher and more variable ΔHue values were recorded for *Pseudomonas putida* ATCC 33015/PCM 2152. *Pseudomonas aeruginosa* ATCC 27853 also showed comparatively higher ΔHue values than several other rod-shaped strains. In coccal bacteria, the highest, least negative ΔHue values were observed for *Enterococcus faecalis* ATCC 29212, *Enterococcus faecium* PCM 1859, *Streptococcus pyogenes* ATCC 12344, and *Streptococcus sanguinis* ATCC 10556. Lower median values and broader distributions were observed particularly for *Staphylococcus aureus* ATCC 6538P and *Staphylococcus xylosus* ATCC 700404.

Although statistically significant inter-strain differences were demonstrated, partial overlap of ΔHue ranges was observed in both morphological groups. This overlap was particularly evident among strains with intermediate ΔHue values, where the distributions were not clearly separated. Therefore, statistically significant differences in ΔHue should not be interpreted as evidence of unequivocal strain discrimination. Instead, ΔHue may provide information on relative strain-associated and temporal fluorescence response patterns within the present dataset. When data from the entire incubation period were considered together, temporal variability broadened the within-strain distributions and reduced separation between some strains.

These findings support H1, as significant differences between bacterial strains were observed after correction for the agar background. Nevertheless, the partial overlap between several ΔHue distributions indicates that this parameter alone is insufficient for unequivocal bacterial identification and should not be considered a standalone diagnostic marker. Instead, ΔHue should be interpreted as an exploratory quantitative image-derived parameter for within-dataset monitoring of fluorescence colour changes. Its analytical performance may potentially be improved by integration with complementary features, including fluorescence intensity, saturation, brightness, texture, spatial signal distribution, and colony morphology.

### 3.5. Qualitative Assessment

To complement the quantitative ΔHue analysis, the acquired images were visually assessed for signal presence, perceived intensity, colour appearance, spatial distribution, and temporal changes. Qualitative visual assessment of the recorded fluorescence signal showed that, in both rod-shaped and coccal bacteria, the signal was generally of low visual intensity. In most analysed samples, the emitted fluorescence was close to the threshold of visual perception, which made reliable detection by direct visual observation difficult. As a consequence, precise evaluation of the spatial distribution pattern of the fluorescence signal within individual bacterial colonies was limited.

An exception to this general pattern was observed for two strains that showed clearly visible photoinduced fluorescence. Among coccal bacteria, this was *Staphylococcus aureus* ATCC 6538P (SA65), whereas among rod-shaped bacteria, it was *Proteus mirabilis* PCM 543 (PM). The fluorescence signal observed for *P. mirabilis* was visually stronger than that observed for *S. aureus*. In both strains, an apparent time-dependent increase in visually perceived fluorescence intensity was noted. The signal became progressively more evident at consecutive incubation time points and reached its highest visual intensity at 168 h. In addition, both *S. aureus* ATCC 6538P and *P. mirabilis* PCM 543 showed a characteristic spatial distribution of the fluorescence signal, which was concentrated mainly in the central regions of the colonies and appeared less intense in the peripheral areas.

For the remaining bacterial strains, including both rod-shaped and coccal forms, no clearly visible temporal increase in fluorescence intensity was observed across successive incubation time points. The signal remained at a similarly low and difficult-to-detect level throughout the observation period, which limited the possibility of visually assessing temporal fluorescence changes.

**Figure 8:**
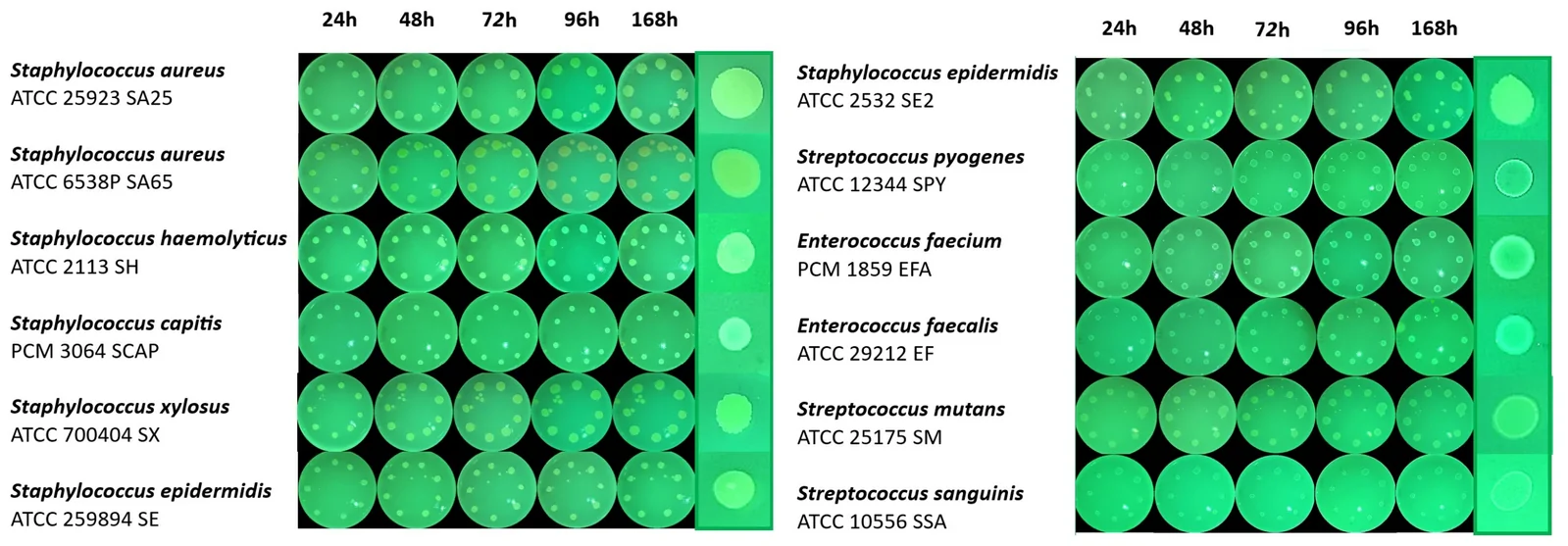
Representative autofluorescence images of coccal bacterial strains under 405 nm laser excitation after 24, 48, 72, 96, and 168 h of incubation. The figure shows strain-specific visual changes in fluorescence appearance over time, including differences in signal intensity and spatial distribution within bacterial colonies.

**Figure 9:**
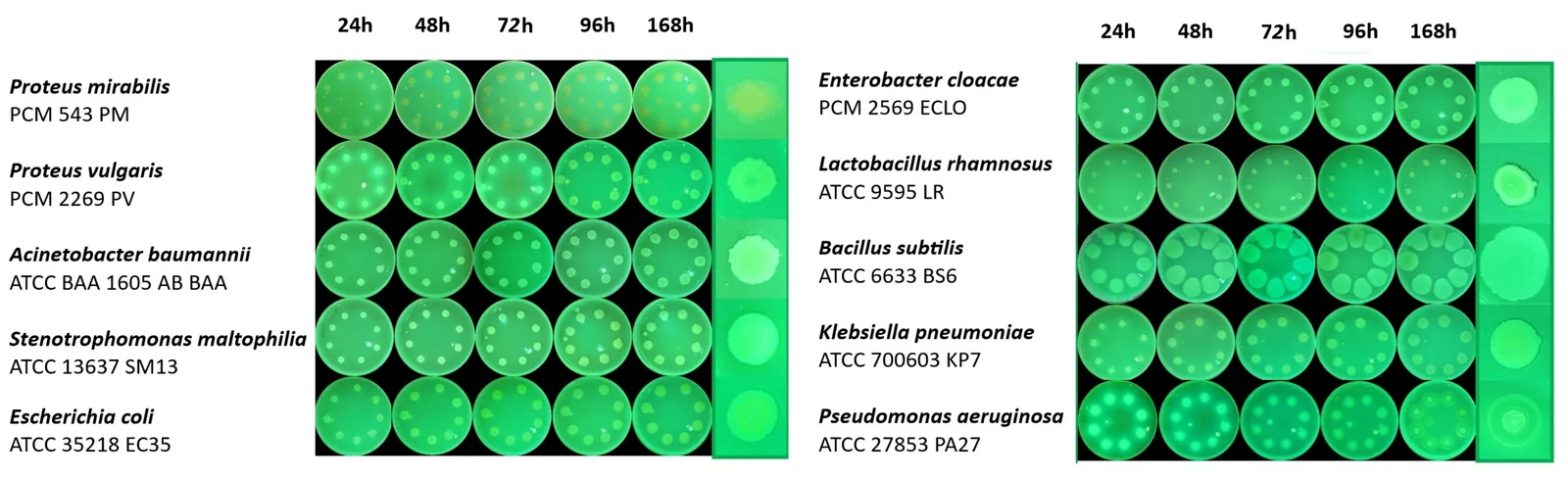
Representative autofluorescence images of rod-shaped bacterial strains under 405 nm laser excitation after 24, 48, 72, 96, and 168 h of incubation. The figure illustrates strain-specific visual differences in autofluorescence intensity and spatial signal distribution during colony development.

Because of the low visual intensity of the recorded fluorescence signal, evaluation of its spatial distribution within bacterial colonies was substantially limited. In most strains, it was not possible to clearly distinguish between diffuse fluorescence distributed across the colony surface and fluorescence concentrated in localized regions. This limitation applied to both rod-shaped and coccal bacteria, with the exception of *S. aureus* ATCC 6538P and *P. mirabilis* PCM 543, in which the higher visual signal intensity allowed a more detailed assessment of its spatial distribution (Figure 8 and Figure 9).

## 4. Discussion

The aim of this study was to investigate whether selected bacterial strains exhibit measurable changes in fluorescence colour under 405 nm laser excitation, whether these changes evolve during cultivation, and whether temporal fluorescence response patterns differ between strains. The temporal profiles demonstrated several distinct patterns of background-corrected fluorescence colour change. Among rod-shaped bacteria, *Stenotrophomonas maltophilia* ATCC 13637 showed a progressive decrease in ΔHue, whereas *Pseudomonas aeruginosa* ATCC 2562 decreased up to 96 h and showed a slight increase at 168 h. *Proteus mirabilis* PCM 543 exhibited pronounced non-linear fluctuations, while *Lactobacillus rhamnosus* ATCC 9595 showed relatively stable early values followed by a transient increase at 96 h. Among coccal bacteria, comparatively stable profiles were observed for *Enterococcus faecalis* ATCC 29212, *Enterococcus faecium* PCM 1859, *Streptococcus pyogenes* ATCC 12344, and *Streptococcus sanguinis* ATCC 10556. In contrast, *Staphylococcus aureus* ATCC 6538P and *Staphylococcus xylosus* ATCC 700404 exhibited pronounced non-linear changes. These contrasting profiles illustrate that the direction and magnitude of ΔHue changes were strain-dependent and could not be represented by a single general temporal pattern. Previous studies on bacterial autofluorescence have focused primarily on signal intensity and on the presence of red fluorescence associated with porphyrins [18,70]. Earlier research demonstrated the possibility of differentiating dental plaque bacteria on the basis of fluorescence intensity ratios under 405 nm excitation [71,72]. It has also been shown that porphyrin fluorescence spectra depend on both species and substrate composition [73,74], and that the autofluorescence intensity of wound-associated bacteria is influenced by species, growth phase, and environmental conditions [24]. Against this background, the present study extends previous approaches by applying background-corrected ΔHue analysis to longitudinal monitoring of fluorescence colour changes across multiple *Candida* strains [75].

The biological basis of the observed fluorescence changes cannot be determined directly from the present data. Under 405 nm excitation, multiple endogenous fluorophores may contribute simultaneously to the detected signal, and their relative contributions may change during colony development [76]. Porphyrins are expected to contribute because they absorb strongly within the Soret band and emit predominantly in the red and red-orange spectral regions. Their abundance depends on bacterial species as well as growth conditions [24,73,77]. In interpreting these findings, it is also important to consider flavins, including FAD and FMN, which emit in the green-yellow range and whose contribution may reflect cellular redox metabolism [78]. NADH and NADPH remain important metabolic fluorophores. Nevertheless, under excitation exclusively with 405 nm light, they should not be assumed to exert a dominant influence on the obtained Hue values without additional spectroscopic analysis [76,77,79]. Certain bacterial species, such as *Pseudomonas aeruginosa*, may additionally exhibit cyan or blue-green fluorescence associated with pyoverdine production [80,81,82]. Because the present study relied on RGB-based Hue analysis without spectral separation or calibration, the measured colour should be interpreted as the integrated fluorescence response detected by the imaging. The statistically significant differences between strains and incubation time points may reflect strain-specific differences in growth kinetics, physiological state, metabolism, and endogenous fluorophore production. Therefore, Hue should be regarded as a quantitative descriptor for monitoring temporal fluorescence colour changes under standardized conditions and not as a direct taxonomic or standalone bacterial identification marker.

The differences observed between the predefined bacterial groups may reflect variations in the overall physiological state of bacterial colonies, including strain-specific metabolic activity, growth kinetics, biomass accumulation, endogenous fluorophore production, oxygen metabolism, and adaptation to the culture environment [83,84,85,86,87,88,89,90,91]. Although Gram-positive and Gram-negative bacteria differ in their cell-envelope organization, the present study does not allow the contribution of cell-wall structure to be separated from other biological factors influencing the recorded fluorescence response. Accordingly, the observed differences should not be interpreted as evidence that peptidoglycan thickness, cell-envelope structure, or bacterial morphology directly determines fluorescence colour. The recorded signal may be influenced by multiple factors, including bacterial strain, growth phase, oxygen availability, nutrient conditions, pH, medium composition, and the production or transformation of endogenous fluorophores [24,77,92]. All strains were cultivated under standardized aerobic conditions using the same culture media, inoculum density, temperature, and incubation time points. Nevertheless, intrinsic differences in metabolic pathways, growth rates, oxygen requirements, physiological state, biomass accumulation, and fluorophore production were not directly quantified. Moreover, Gram-staining status and cellular morphology were not fully independent in the selected strain panel, because all coccal strains were Gram-positive, whereas most rod-shaped strains were Gram-negative. Therefore, the planned contrasts demonstrate differences between the predefined groups represented in this dataset but cannot establish independent causal effects of Gram status or cellular morphology. In this context, the results obtained from monocultures should be regarded as a preliminary characterization of strain-dependent fluorescence responses under controlled laboratory conditions [93,94,95,96,97]. Their verification in single-species and multispecies biofilms appears to be the necessary next stage, particularly because, under clinical conditions in both the oral cavity and chronic wounds, bacteria predominantly exist in the form of biofilms [97,98,99,100]. Moreover, oral bacteria such as *Streptococcus sanguinis* may exert systemic effects beyond the oral cavity, including the induction of vascular inflammation and the progression of atherosclerosis in experimental models [101,102,103,104].

A key feature of this study is the use of the Hue parameter as a quantitative image-derived descriptor of fluorescence colour changes under 405 nm laser excitation. Unlike fluorescence intensity, which is strongly affected by biomass, colony size, exposure conditions, and detector sensitivity, Hue provides a relative description of the dominant colour component within the acquired image. The HSV/HSB colour model enables quantitative assessment of temporal colour variations under fixed imaging conditions. Nonetheless, Hue represents an integrated characteristic of the fluorescence signal and does not provide information about individual spectral components. Under 405 nm excitation, emissions from multiple endogenous fluorophores may overlap, meaning that the measured Hue value reflects the combined fluorescence response detected at a given time point rather than a specific fluorophore or bacterial identity. Therefore, Hue should be considered a parameter for monitoring fluorescence colour changes rather than a standalone diagnostic or taxonomic marker. The use of a consumer smartphone camera offers practical accessibility and has previously been explored for fluorescence-based imaging [72,105,106,107]; however, it also constitutes an important methodological limitation. Although all images were acquired using the same device, yellow optical filter, camera-to-sample distance, and acquisition geometry, proprietary image-signal processing could not be completely disabled or independently controlled. In particular, automatic white-balance adjustment, tone mapping, computational image enhancement, and device-specific sensor characteristics may affect the recorded RGB values and, consequently, the calculated Hue parameter. Moreover, the images were acquired in JPEG rather than RAW/DNG format, and no physical colour-calibration target or calibrated fluorescence reference standard was used. Therefore, the obtained Hue values are device-, software-, and protocol-dependent image-derived parameters and should not be interpreted as absolute colourimetric measurements. They cannot be assumed to be directly transferable between different smartphones, camera applications, operating-system versions, or imaging sessions. The present findings should consequently be limited to relative within-dataset comparisons obtained using the same imaging system under fixed external acquisition conditions. Because the bacterial fluorescence signal was generally weak, optical contributions from the agar substrate may also have influenced the measured colour values, particularly in thin colonies and near colony boundaries. This influence may vary with colony biomass, thickness, cell density, and optical opacity during incubation. Future studies should incorporate multiple local background ROIs, colony-specific annular background correction, or pixel-level background modelling to better account for spatial variation across the agar plate. Integrating Hue with additional image-derived parameters and spectrally resolved measurements may further improve the robustness and biological interpretability of the proposed approach.

An important observation from the qualitative assessment was the relatively low visual intensity of the autofluorescence signal emitted by the examined bacterial colonies. In both rod-shaped and coccal bacteria, autofluorescence was, in most cases, close to the threshold of visual perception, which made its unequivocal detection difficult without the support of quantitative image analysis. This finding is relevant in the context of our previous work on fungal autofluorescence, in which Candida spp., including both *Candida albicans* and non-*albicans Candida* species, showed a more readily visible autofluorescence signal under comparable violet-light excitation conditions [72]. Although the present study was not designed to directly compare bacterial and fungal fluorescence responses, this difference suggests that the visibility of fluorescence depends not only on imaging conditions but also on the biological characteristics of the examined microorganisms, including the composition and relative abundance of endogenous fluorophores and the optical properties of microbial colonies. Importantly, the qualitative observations reported here should not be interpreted as direct measures of fluorescence intensity, as the quantitative analysis in the present study was based on the background-corrected ΔHue parameter.

A significant constraint of this investigation is its in vitro design. Analyzing isolated strains under controlled conditions enables the detection of inter-bacterial differences and the assessment of temporal photoinduced fluorescence colour changes, but it does not reflect the complexity of the conditions present in the human body [106,107,108]. In the oral cavity and in chronic wounds, bacteria exist within multispecies biofilms and in the presence of host proteins, inflammatory cells, saliva or exudate, fluctuating pH, oxygen gradients, and metabolic products generated by other microorganisms [66,109,110,111,112,113,114,115,116]. All of these factors may influence both the production of endogenous fluorophores and the recorded autofluorescence colour [117]. Furthermore, exposure to 405 nm violet light may influence bacterial physiology and fluorophore composition through endogenous photosensitization and photooxidative stress [14,15,16,17,77]. Potential effects include photobleaching, altered metabolic activity, changes in endogenous fluorophore profiles, adaptive stress responses, and reduced bacterial viability. Therefore, temporal Hue changes should not be attributed exclusively to colony ageing or growth-phase progression. Because non-irradiated controls, viability assays, reactive oxygen species measurements, and spectroscopic analyses were not included, the observed changes may reflect both colony development and photoinduced effects. Moreover, Hue represents an integrated optical signal and does not allow identification or quantification of individual fluorophores. Further studies should include non-irradiated controls, dose–response experiments, viability assessment, spectroscopic analysis, and validation in larger strain panels and biofilm models. Integration of Hue with fluorescence intensity, saturation, brightness, texture, spatial signal distribution, and colony morphology may improve analytical performance. Although the smartphone-based system offers practical accessibility [72,105,106,107], its quantitative applicability is limited by JPEG acquisition, automatic white balance, proprietary image processing, lack of RAW/DNG files and colour calibration, and possible agar-background contributions. The obtained Hue values should therefore be regarded as device-, software-, session-, and protocol-dependent parameters suitable for relative within-dataset comparisons rather than absolute colourimetric measurements. At present, the method should be considered an exploratory tool for controlled in vitro fluorescence monitoring and not a standalone diagnostic method [118,119,120,121].

## 5. Conclusions

This study demonstrates that the background-corrected, image-derived Hue parameter, ΔHue = Hmean − Hmean_BG, changed significantly across incubation time points and differed between the analysed bacterial strains under the applied 405 nm violet-light imaging protocol. Significant strain × time interactions in both rod-shaped and coccal bacteria showed that the direction and magnitude of temporal ΔHue changes were strain-dependent and could not be represented by a single general response pattern.

The principal methodological contribution of this study is the application of background-corrected ΔHue analysis as a quantitative descriptor for relative within-dataset monitoring of temporal fluorescence colour changes. The developed approach combines standardized 405 nm excitation, fixed smartphone-based image acquisition, agar-background correction, and accessible digital image analysis. It may therefore provide a simple and low-cost framework for exploratory in vitro monitoring of bacterial fluorescence responses, particularly when subtle colour changes are difficult to assess visually.

However, ΔHue represents an integrated optical parameter influenced by multiple endogenous fluorophores, colony development, repeated light exposure, agar-background characteristics, and device-level image processing. The substantial overlap between several strain distributions, together with the absence of spectral resolution, RAW acquisition, and colour calibration, precludes the use of ΔHue as a standalone parameter for bacterial identification, Gram-group differentiation, or clinical diagnosis. Further validation using calibrated imaging, non-irradiated controls, viability assays, spectrally resolved measurements, and additional image-derived parameters is required to establish the reproducibility and broader analytical applicability of the proposed method.

## Figures and Tables

**Table 1:** Reference rod-shaped bacterial strains used in the study.

Species	Collection No.	Strain Code	Gram Staining
*Klebsiella pneumoniae*	ATCC 700603	KP7	Gram-negative
*Escherichia coli*	ATCC 25922	EC25	Gram-negative
*Escherichia coli*	ATCC 35218	EC35	Gram-negative
*Enterobacter cloacae*	PCM 2569	ECLO	Gram-negative
*Proteus mirabilis*	PCM 543	PM	Gram-negative
*Proteus vulgaris*	PCM 2269	PV	Gram-negative
*Pseudomonas aeruginosa*	ATCC 27853	PA27	Gram-negative
*Pseudomonas aeruginosa*	ATCC 2562	PA25	Gram-negative
*Pseudomonas putida*	ATCC 33015/PCM 2152	PP	Gram-negative
*Acinetobacter baumannii*	ATCC 2740	AB27	Gram-negative
*Acinetobacter baumannii*	ATCC BAA 1605	AB_BAA	Gram-negative
*Stenotrophomonas maltophilia*	ATCC 13637	SM13	Gram-negative
*Lactobacillus rhamnosus*	ATCC 9595	LR	Gram-positive
*Bacillus subtilis*	ATCC 6633	BS	Gram-positive

**Table 2:** Reference coccal bacterial strains used in the study.

Species	Collection No.	Strain Code	Gram Staining
*Staphylococcus aureus*	ATCC 25923	SA25	Gram-positive
*Staphylococcus aureus*	ATCC 6538P	SA65	Gram-positive
*Staphylococcus haemolyticus*	ATCC 2113	SH	Gram-positive
*Staphylococcus epidermidis*	ATCC 25984	SE	Gram-positive
*Staphylococcus epidermidis*	ATCC 2479	SE1	Gram-positive
*Staphylococcus epidermidis*	ATCC 2532	SE2	Gram-positive
*Staphylococcus xylosus*	ATCC 700404	SX	Gram-positive
*Staphylococcus capitis*	PCM 3064	SCAP	Gram-positive
*Enterococcus faecalis*	ATCC 29212	EF	Gram-positive
*Enterococcus faecium*	PCM 1859	EFA	Gram-positive
*Streptococcus pyogenes*	ATCC 12344	SPY	Gram-positive
*Streptococcus mutans*	ATCC 25175	SM	Gram-positive
*Streptococcus agalactiae*	PCM 2683	SAG	Gram-positive
*Streptococcus sanguinis*	ATCC 10556	SSA	Gram-positive

**Table 3:** Results of ART-ANOVA for the effects of bacterial strain, incubation time, and their interaction on background-corrected Hue values (ΔHue = Hmean − Hmean_BG) in rod-shaped bacteria.

Effect	Df	Df.res	F Value	*p*-Value
Strain	13	490	398.22	<0.001
Time_h	4	490	419.95	<0.001
Strain × Time_h	52	490	44.74	<0.001

**Table 4:** Results of ART-ANOVA for the effects of bacterial strain, incubation time, and their interaction on background-corrected Hue values (ΔHue = Hmean − Hmean_BG) in coccal bacteria.

Effect	Df	Residual Df	F Value	*p*-Value
Strain	13	490	323.712	<0.001
Time_h	4	490	85.452	<0.001
Strain × Time_h	52	490	25.375	<0.001

**Table 5:** Exploratory planned ART contrasts for differences in background-corrected Hue values (ΔHue = Hmean − Hmean_BG) between the predefined Gram-staining groups and between rod-shaped and coccal bacteria.

Comparison	Estimate	SE	df	t Ratio	*p* Value
Gram-positive vs. Gram-negative	73.5	19.4	1110	3.788	0.0002
Rod-shaped bacteria vs. coccal bacteria	−86.3	19.2	1110	−4.503	<0.0001

## Data Availability

The original contributions presented in this study are included in the article/Supplementary Materials. Further inquiries can be directed to the corresponding authors.

## Supplementary Materials

The following supporting information can be downloaded at: https://www.mdpi.com/article/10.3390/mi17080885/s1, Table S1: Pairwise comparisons of rod-shaped bacterial strains at 24 h based on mean hue values (ART post hoc test with Holm correction); Table S2: Pairwise comparisons of rod-shaped bacterial strains at 48 h based on mean hue values (ART post hoc test with Holm correction); Table S3: Pairwise comparisons of rod-shaped bacterial strains at 72 h based on mean hue values (ART post hoc test with Holm correction); Table S4: Pairwise comparisons of rod-shaped bacterial strains at 96 h based on mean hue values (ART post hoc test with Holm correction); Table S5: Pairwise comparisons of rod-shaped bacterial strains at 168 h based on mean hue values (ART post hoc test with Holm correction); Table S6: Pairwise comparisons of cocci-shaped bacterial strains at 24 h based on mean hue values (ART post hoc test with Holm correction); Table S7: Pairwise comparisons of cocci-shaped bacterial strains at 48 h based on mean hue values (ART post hoc test with Holm correction); Table S8: Pairwise comparisons of cocci-shaped bacterial strains at 72 h based on mean hue values (ART post hoc test with Holm correction); Table S9: Pairwise comparisons of cocci-shaped bacterial strains at 96 h based on mean hue values (ART post hoc test with Holm correction); Table S10: Pairwise comparisons of cocci-shaped bacterial strains at 168 h based on mean hue values (ART post hoc test with Holm correction); Table S11: Statistical workflow and R functions applied in the analysis of bacterial hue values; Figure S1: Boxplots used for visual assessment of outliers in bacilli strains; Figure S2: Boxplots used for visual assessment of outliers in coccal bacterial strains.
