# Supplementary material for: Photoinduced Fluorescence Changes in Selected Bacterial Strains Under 405 nm Laser Excitation: An In Vitro Hue-Based Monitoring Approach
Source: Micromachines (Basel). 2026 Jul 24;17(8):885. doi: 10.3390/mi17080885 (PMC13515274; doi:10.3390/mi17080885)
Supplement: Supplementary file 1 [file micromachines-17-00885-s001.zip › micromachines-4407006-supplementary.pdf]

Supplementary materials

Table S1. Pairwise comparisons of rod-shaped bacterial strains at 24 h based on mean hue values (ART post hoc test with Holm correction).

Comparison	Estimate	Holm-adjusted p-value
AB_BAA vs AB27	-21.12	<0.001
AB_BAA vs BS	31.25	<0.001
AB_BAA vs EC25	24.62	<0.001
AB_BAA vs EC35	-17.88	<0.001
AB_BAA vs ECLO	9.25	0.009
AB_BAA vs KP7	-32.62	<0.001
AB_BAA vs LR	35.62	<0.001
AB_BAA vs PA25	-51.62	<0.001
AB_BAA vs PA27	-41.50	<0.001
AB_BAA vs PM	46.50	<0.001
AB_BAA vs PP	-55.38	<0.001
AB_BAA vs PV	-14.38	<0.001
AB_BAA vs SM13	10.25	0.003
AB27 vs BS	52.38	<0.001
AB27 vs EC25	45.75	<0.001
AB27 vs EC35	3.25	0.682
AB27 vs ECLO	30.38	<0.001
AB27 vs KP7	-11.50	<0.001
AB27 vs LR	56.75	<0.001
AB27 vs PA25	-30.50	<0.001
AB27 vs PA27	-20.38	<0.001
AB27 vs PM	67.62	<0.001
AB27 vs PP	-34.25	<0.001
AB27 vs PV	6.75	0.103
AB27 vs SM13	31.38	<0.001
BS vs EC25	-6.62	0.103
BS vs EC35	-49.12	<0.001
BS vs ECLO	-22.00	<0.001
BS vs KP7	-63.88	<0.001
BS vs LR	4.38	0.552
BS vs PA25	-82.88	<0.001
BS vs PA27	-72.75	<0.001
BS vs PM	15.25	<0.001
BS vs PP	-86.62	<0.001
BS vs PV	-45.62	<0.001
BS vs SM13	-21.00	<0.001
EC25 vs EC35	-42.50	<0.001
EC25 vs ECLO	-15.38	<0.001
EC25 vs KP7	-57.25	<0.001
EC25 vs LR	11.00	0.001

Comparison	Estimate	Holm-adjusted p-value
EC25 vs PA25	-76.25	<0.001
EC25 vs PA27	-66.12	<0.001
EC25 vs PM	21.88	<0.001
EC25 vs PP	-80.00	<0.001
EC25 vs PV	-39.00	<0.001
EC25 vs SM13	-14.38	<0.001
EC35 vs ECLO	27.12	<0.001
EC35 vs KP7	-14.75	<0.001
EC35 vs LR	53.50	<0.001
EC35 vs PA25	-33.75	<0.001
EC35 vs PA27	-23.62	<0.001
EC35 vs PM	64.38	<0.001
EC35 vs PP	-37.50	<0.001
EC35 vs PV	3.50	0.682
EC35 vs SM13	28.12	<0.001
ECLO vs KP7	-41.88	<0.001
ECLO vs LR	26.38	<0.001
ECLO vs PA25	-60.88	<0.001
ECLO vs PA27	-50.75	<0.001
ECLO vs PM	37.25	<0.001
ECLO vs PP	-64.62	<0.001
ECLO vs PV	-23.62	<0.001
ECLO vs SM13	1.00	0.714
KP7 vs LR	68.25	<0.001
KP7 vs PA25	-19.00	<0.001
KP7 vs PA27	-8.88	0.012
KP7 vs PM	79.12	<0.001
KP7 vs PP	-22.75	<0.001
KP7 vs PV	18.25	<0.001
KP7 vs SM13	42.88	<0.001
LR vs PA25	-87.25	<0.001
LR vs PA27	-77.12	<0.001
LR vs PM	10.88	0.002
LR vs PP	-91.00	<0.001
LR vs PV	-50.00	<0.001
LR vs SM13	-25.38	<0.001
PA25 vs PA27	10.12	0.003
PA25 vs PM	98.12	<0.001
PA25 vs PP	-3.75	0.682
PA25 vs PV	37.25	<0.001
PA25 vs SM13	61.88	<0.001
PA27 vs PM	88.00	<0.001
PA27 vs PP	-13.88	<0.001

Comparison	Estimate	Holm-adjusted p-value
PA27 vs PV	27.12	<0.001
PA27 vs SM13	51.75	<0.001
PM vs PP	-101.88	<0.001
PM vs PV	-60.88	<0.001
PM vs SM13	-36.25	<0.001
PP vs PV	41.00	<0.001
PP vs SM13	65.62	<0.001
PV vs SM13	24.62	<0.001

Table S2. Pairwise comparisons of rod-shaped bacterial strains at 48 h based on mean hue values (ART post hoc test with Holm correction).

Comparison	Estimate	Holm-adjusted p-value
AB_BAA vs AB27	-22.125	<0.001
AB_BAA vs BS	58.000	<0.001
AB_BAA vs EC25	59.375	<0.001
AB_BAA vs EC35	41.875	<0.001
AB_BAA vs ECLO	17.688	<0.001
AB_BAA vs KP7	22.188	<0.001
AB_BAA vs LR	70.625	<0.001
AB_BAA vs PA25	3.375	1.000
AB_BAA vs PA27	-14.000	0.004
AB_BAA vs PM	59.500	<0.001
AB_BAA vs PP	-30.125	<0.001
AB_BAA vs PV	7.625	0.276
AB_BAA vs SM13	32.250	<0.001
AB27 vs BS	80.125	<0.001
AB27 vs EC25	81.500	<0.001
AB27 vs EC35	64.000	<0.001
AB27 vs ECLO	39.812	<0.001
AB27 vs KP7	44.312	<0.001
AB27 vs LR	92.750	<0.001
AB27 vs PA25	25.500	<0.001
AB27 vs PA27	8.125	0.256
AB27 vs PM	81.625	<0.001
AB27 vs PP	-8.000	0.256
AB27 vs PV	29.750	<0.001
AB27 vs SM13	54.375	<0.001
BS vs EC25	1.375	1.000
BS vs EC35	-16.125	<0.001
BS vs ECLO	-40.312	<0.001
BS vs KP7	-35.812	<0.001
BS vs LR	12.625	0.012
BS vs PA25	-54.625	<0.001

Comparison	Estimate	Holm-adjusted p-value
BS vs PA27	-72.000	<0.001
BS vs PM	1.500	1.000
BS vs PP	-88.125	<0.001
BS vs PV	-50.375	<0.001
BS vs SM13	-25.750	<0.001
EC25 vs EC35	-17.500	<0.001
EC25 vs ECLO	-41.688	<0.001
EC25 vs KP7	-37.188	<0.001
EC25 vs LR	11.250	0.038
EC25 vs PA25	-56.000	<0.001
EC25 vs PA27	-73.375	<0.001
EC25 vs PM	0.125	1.000
EC25 vs PP	-89.500	<0.001
EC25 vs PV	-51.750	<0.001
EC25 vs SM13	-27.125	<0.001
EC35 vs ECLO	-24.188	<0.001
EC35 vs KP7	-19.688	<0.001
EC35 vs LR	28.750	<0.001
EC35 vs PA25	-38.500	<0.001
EC35 vs PA27	-55.875	<0.001
EC35 vs PM	17.625	<0.001
EC35 vs PP	-72.000	<0.001
EC35 vs PV	-34.250	<0.001
EC35 vs SM13	-9.625	0.098
ECLO vs KP7	4.500	1.000
ECLO vs LR	52.938	<0.001
ECLO vs PA25	-14.312	0.003
ECLO vs PA27	-31.688	<0.001
ECLO vs PM	41.812	<0.001
ECLO vs PP	-47.812	<0.001
ECLO vs PV	-10.062	0.084
ECLO vs SM13	14.562	0.003
KP7 vs LR	48.438	<0.001
KP7 vs PA25	-18.812	<0.001
KP7 vs PA27	-36.188	<0.001
KP7 vs PM	37.312	<0.001
KP7 vs PP	-52.312	<0.001
KP7 vs PV	-14.562	0.003
KP7 vs SM13	10.062	0.084
LR vs PA25	-67.250	<0.001
LR vs PA27	-84.625	<0.001
LR vs PM	-11.125	0.039
LR vs PP	-100.750	<0.001

Comparison	Estimate	Holm-adjusted p-value
LR vs PV	-63.000	<0.001
LR vs SM13	-38.375	<0.001
PA25 vs PA27	-17.375	<0.001
PA25 vs PM	56.125	<0.001
PA25 vs PP	-33.500	<0.001
PA25 vs PV	4.250	1.000
PA25 vs SM13	28.875	<0.001
PA27 vs PM	73.500	<0.001
PA27 vs PP	-16.125	<0.001
PA27 vs PV	21.625	<0.001
PA27 vs SM13	46.250	<0.001
PM vs PP	-89.625	<0.001
PM vs PV	-51.875	<0.001
PM vs SM13	-27.250	<0.001
PP vs PV	37.750	<0.001
PP vs SM13	62.375	<0.001
PV vs SM13	24.625	<0.001

Table S3. Pairwise comparisons of rod-shaped bacterial strains at 72 h based on mean hue values (ART post hoc test with Holm correction).

Comparison	Estimate	Holm-adjusted p-value
AB_BAA vs AB27	33.75	<0.001
AB_BAA vs BS	-19.12	<0.001
AB_BAA vs EC25	45.75	<0.001
AB_BAA vs EC35	8.38	0.018
AB_BAA vs ECLO	64.50	<0.001
AB_BAA vs KP7	-5.75	0.151
AB_BAA vs LR	58.75	<0.001
AB_BAA vs PA25	25.38	<0.001
AB_BAA vs PA27	19.50	<0.001
AB_BAA vs PM	81.62	<0.001
AB_BAA vs PP	-15.38	<0.001
AB_BAA vs PV	43.75	<0.001
AB_BAA vs SM13	73.62	<0.001
AB27 vs BS	-52.88	<0.001
AB27 vs EC25	12.00	<0.001
AB27 vs EC35	-25.38	<0.001
AB27 vs ECLO	30.75	<0.001
AB27 vs KP7	-39.50	<0.001
AB27 vs LR	25.00	<0.001
AB27 vs PA25	-8.38	0.018
AB27 vs PA27	-14.25	<0.001
AB27 vs PM	47.88	<0.001

Comparison	Estimate	Holm-adjusted p-value
AB27 vs PP	-49.12	<0.001
AB27 vs PV	10.00	0.003
AB27 vs SM13	39.88	<0.001
BS vs EC25	64.88	<0.001
BS vs EC35	27.50	<0.001
BS vs ECLO	83.62	<0.001
BS vs KP7	13.38	<0.001
BS vs LR	77.88	<0.001
BS vs PA25	44.50	<0.001
BS vs PA27	38.62	<0.001
BS vs PM	100.75	<0.001
BS vs PP	3.75	0.327
BS vs PV	62.88	<0.001
BS vs SM13	92.75	<0.001
EC25 vs EC35	-37.38	<0.001
EC25 vs ECLO	18.75	<0.001
EC25 vs KP7	-51.50	<0.001
EC25 vs LR	13.00	<0.001
EC25 vs PA25	-20.38	<0.001
EC25 vs PA27	-26.25	<0.001
EC25 vs PM	35.88	<0.001
EC25 vs PP	-61.12	<0.001
EC25 vs PV	-2.00	0.456
EC25 vs SM13	27.88	<0.001
EC35 vs ECLO	56.12	<0.001
EC35 vs KP7	-14.12	<0.001
EC35 vs LR	50.38	<0.001
EC35 vs PA25	17.00	<0.001
EC35 vs PA27	11.12	0.001
EC35 vs PM	73.25	<0.001
EC35 vs PP	-23.75	<0.001
EC35 vs PV	35.38	<0.001
EC35 vs SM13	65.25	<0.001
ECLO vs KP7	-70.25	<0.001
ECLO vs LR	-5.75	0.151
ECLO vs PA25	-39.12	<0.001
ECLO vs PA27	-45.00	<0.001
ECLO vs PM	17.12	<0.001
ECLO vs PP	-79.88	<0.001
ECLO vs PV	-20.75	<0.001
ECLO vs SM13	9.12	0.008
KP7 vs LR	64.50	<0.001
KP7 vs PA25	31.12	<0.001

Comparison	Estimate	Holm-adjusted p-value
KP7 vs PA27	25.25	<0.001
KP7 vs PM	87.38	<0.001
KP7 vs PP	-9.62	0.005
KP7 vs PV	49.50	<0.001
KP7 vs SM13	79.38	<0.001
LR vs PA25	-33.38	<0.001
LR vs PA27	-39.25	<0.001
LR vs PM	22.88	<0.001
LR vs PP	-74.12	<0.001
LR vs PV	-15.00	<0.001
LR vs SM13	14.88	<0.001
PA25 vs PA27	-5.88	0.151
PA25 vs PM	56.25	<0.001
PA25 vs PP	-40.75	<0.001
PA25 vs PV	18.38	<0.001
PA25 vs SM13	48.25	<0.001
PA27 vs PM	62.12	<0.001
PA27 vs PP	-34.88	<0.001
PA27 vs PV	24.25	<0.001
PA27 vs SM13	54.12	<0.001
PM vs PP	-97.00	<0.001
PM vs PV	-37.88	<0.001
PM vs SM13	-8.00	0.021
PP vs PV	59.12	<0.001
PP vs SM13	89.00	<0.001
PV vs SM13	29.88	<0.001

Table S4. Pairwise comparisons of rod-shaped bacterial strains at 96 h based on mean hue values (ART post hoc test with Holm correction).

Comparison	Estimate	Holm-adjusted p-value
AB_BAA vs AB27	8.000	0.0008
AB_BAA vs BS	-38.750	<0.0001
AB_BAA vs EC25	41.250	<0.0001
AB_BAA vs EC35	45.375	<0.0001
AB_BAA vs ECLO	28.375	<0.0001
AB_BAA vs KP7	-16.000	<0.0001
AB_BAA vs LR	-33.250	<0.0001
AB_BAA vs PA25	29.000	<0.0001
AB_BAA vs PA27	-24.000	<0.0001
AB_BAA vs PM	64.000	<0.0001
AB_BAA vs PP	16.000	<0.0001
AB_BAA vs PV	-8.000	0.0008
AB_BAA vs SM13	56.000	<0.0001

Comparison	Estimate	Holm-adjusted p-value
AB27 vs BS	-46.750	<0.0001
AB27 vs EC25	33.250	<0.0001
AB27 vs EC35	37.375	<0.0001
AB27 vs ECLO	20.375	<0.0001
AB27 vs KP7	-24.000	<0.0001
AB27 vs LR	-41.250	<0.0001
AB27 vs PA25	21.000	<0.0001
AB27 vs PA27	-32.000	<0.0001
AB27 vs PM	56.000	<0.0001
AB27 vs PP	8.000	0.0008
AB27 vs PV	-16.000	<0.0001
AB27 vs SM13	48.000	<0.0001
BS vs EC25	80.000	<0.0001
BS vs EC35	84.125	<0.0001
BS vs ECLO	67.125	<0.0001
BS vs KP7	22.750	<0.0001
BS vs LR	5.500	0.0180
BS vs PA25	67.750	<0.0001
BS vs PA27	14.750	<0.0001
BS vs PM	102.750	<0.0001
BS vs PP	54.750	<0.0001
BS vs PV	30.750	<0.0001
BS vs SM13	94.750	<0.0001
EC25 vs EC35	4.125	0.0753
EC25 vs ECLO	-12.875	<0.0001
EC25 vs KP7	-57.250	<0.0001
EC25 vs LR	-74.500	<0.0001
EC25 vs PA25	-12.250	<0.0001
EC25 vs PA27	-65.250	<0.0001
EC25 vs PM	22.750	<0.0001
EC25 vs PP	-25.250	<0.0001
EC25 vs PV	-49.250	<0.0001
EC25 vs SM13	14.750	<0.0001
EC35 vs ECLO	-17.000	<0.0001
EC35 vs KP7	-61.375	<0.0001
EC35 vs LR	-78.625	<0.0001
EC35 vs PA25	-16.375	<0.0001
EC35 vs PA27	-69.375	<0.0001
EC35 vs PM	18.625	<0.0001
EC35 vs PP	-29.375	<0.0001
EC35 vs PV	-53.375	<0.0001
EC35 vs SM13	10.625	<0.0001
ECLO vs KP7	-44.375	<0.0001

Comparison	Estimate	Holm-adjusted p-value
ECLO vs LR	-61.625	<0.0001
ECLO vs PA25	0.625	0.7502
ECLO vs PA27	-52.375	<0.0001
ECLO vs PM	35.625	<0.0001
ECLO vs PP	-12.375	<0.0001
ECLO vs PV	-36.375	<0.0001
ECLO vs SM13	27.625	<0.0001
KP7 vs LR	-17.250	<0.0001
KP7 vs PA25	45.000	<0.0001
KP7 vs PA27	-8.000	0.0008
KP7 vs PM	80.000	<0.0001
KP7 vs PP	32.000	<0.0001
KP7 vs PV	8.000	0.0008
KP7 vs SM13	72.000	<0.0001
LR vs PA25	62.250	<0.0001
LR vs PA27	9.250	<0.0001
LR vs PM	97.250	<0.0001
LR vs PP	49.250	<0.0001
LR vs PV	25.250	<0.0001
LR vs SM13	89.250	<0.0001
PA25 vs PA27	-53.000	<0.0001
PA25 vs PM	35.000	<0.0001
PA25 vs PP	-13.000	<0.0001
PA25 vs PV	-37.000	<0.0001
PA25 vs SM13	27.000	<0.0001
PA27 vs PM	88.000	<0.0001
PA27 vs PP	40.000	<0.0001
PA27 vs PV	16.000	<0.0001
PA27 vs SM13	80.000	<0.0001
PM vs PP	-48.000	<0.0001
PM vs PV	-72.000	<0.0001
PM vs SM13	-8.000	0.0008
PP vs PV	-24.000	<0.0001
PP vs SM13	40.000	<0.0001
PV vs SM13	64.000	<0.0001

Table S5. Pairwise comparisons of rod-shaped bacterial strains at 168 h based on mean hue values (ART post hoc test with Holm correction).

Comparison	Estimate	Holm-adjusted p-value
AB_BAA vs AB27	-76.625	<0.001
AB_BAA vs BS	-6.500	0.161
AB_BAA vs EC25	-17.125	<0.001
AB_BAA vs EC35	-29.875	<0.001

Comparison	Estimate	Holm-adjusted p-value
AB_BAA vs ECLO	-26.000	<0.001
AB_BAA vs KP7	-44.875	<0.001
AB_BAA vs LR	-77.375	<0.001
AB_BAA vs PA25	1.500	1.000
AB_BAA vs PA27	-62.500	<0.001
AB_BAA vs PM	-89.000	<0.001
AB_BAA vs PP	-58.875	<0.001
AB_BAA vs PV	-45.750	<0.001
AB_BAA vs SM13	15.000	<0.001
AB27 vs BS	70.125	<0.001
AB27 vs EC25	59.500	<0.001
AB27 vs EC35	46.750	<0.001
AB27 vs ECLO	50.625	<0.001
AB27 vs KP7	31.750	<0.001
AB27 vs LR	-0.750	1.000
AB27 vs PA25	78.125	<0.001
AB27 vs PA27	14.125	<0.001
AB27 vs PM	-12.375	<0.001
AB27 vs PP	17.750	<0.001
AB27 vs PV	30.875	<0.001
AB27 vs SM13	91.625	<0.001
BS vs EC25	-10.625	0.003
BS vs EC35	-23.375	<0.001
BS vs ECLO	-19.500	<0.001
BS vs KP7	-38.375	<0.001
BS vs LR	-70.875	<0.001
BS vs PA25	8.000	0.047
BS vs PA27	-56.000	<0.001
BS vs PM	-82.500	<0.001
BS vs PP	-52.375	<0.001
BS vs PV	-39.250	<0.001
BS vs SM13	21.500	<0.001
EC25 vs EC35	-12.750	<0.001
EC25 vs ECLO	-8.875	0.022
EC25 vs KP7	-27.750	<0.001
EC25 vs LR	-60.250	<0.001
EC25 vs PA25	18.625	<0.001
EC25 vs PA27	-45.375	<0.001
EC25 vs PM	-71.875	<0.001
EC25 vs PP	-41.750	<0.001
EC25 vs PV	-28.625	<0.001
EC25 vs SM13	32.125	<0.001
EC35 vs ECLO	3.875	0.916

Comparison	Estimate	Holm-adjusted p-value
EC35 vs KP7	-15.000	<0.001
EC35 vs LR	-47.500	<0.001
EC35 vs PA25	31.375	<0.001
EC35 vs PA27	-32.625	<0.001
EC35 vs PM	-59.125	<0.001
EC35 vs PP	-29.000	<0.001
EC35 vs PV	-15.875	<0.001
EC35 vs SM13	44.875	<0.001
ECLO vs KP7	-18.875	<0.001
ECLO vs LR	-51.375	<0.001
ECLO vs PA25	27.500	<0.001
ECLO vs PA27	-36.500	<0.001
ECLO vs PM	-63.000	<0.001
ECLO vs PP	-32.875	<0.001
ECLO vs PV	-19.750	<0.001
ECLO vs SM13	41.000	<0.001
KP7 vs LR	-32.500	<0.001
KP7 vs PA25	46.375	<0.001
KP7 vs PA27	-17.625	<0.001
KP7 vs PM	-44.125	<0.001
KP7 vs PP	-14.000	<0.001
KP7 vs PV	-0.875	1.000
KP7 vs SM13	59.875	<0.001
LR vs PA25	78.875	<0.001
LR vs PA27	14.875	<0.001
LR vs PM	-11.625	0.001
LR vs PP	18.500	<0.001
LR vs PV	31.625	<0.001
LR vs SM13	92.375	<0.001
PA25 vs PA27	-64.000	<0.001
PA25 vs PM	-90.500	<0.001
PA25 vs PP	-60.375	<0.001
PA25 vs PV	-47.250	<0.001
PA25 vs SM13	13.500	<0.001
PA27 vs PM	-26.500	<0.001
PA27 vs PP	3.625	0.916
PA27 vs PV	16.750	<0.001
PA27 vs SM13	77.500	<0.001
PM vs PP	30.125	<0.001
PM vs PV	43.250	<0.001
PM vs SM13	104.000	<0.001
PP vs PV	13.125	<0.001
PP vs SM13	73.875	<0.001

Comparison	Estimate	Holm-adjusted p-value
PV vs SM13	60.750	<0.001

Table S6. Pairwise comparisons of cocci-shaped bacterial strains at 24 h based on mean hue values (ART post hoc test with Holm correction).

Comparison	Estimate	Holm-adjusted p-value
EF vs EFA	10.88	<0.001
EF vs SA25	54.50	<0.001
EF vs SA65	64.50	<0.001
EF vs SAG	-31.50	<0.001
EF vs SCAP	32.50	<0.001
EF vs SE	47.00	<0.001
EF vs SE1	14.12	<0.001
EF vs SE2	-9.38	0.0002
EF vs SH	24.50	<0.001
EF vs SM	-39.50	<0.001
EF vs SPY	-23.00	<0.001
EF vs SSA	-13.62	<0.001
EF vs SX	44.00	<0.001
EFA vs SA25	43.62	<0.001
EFA vs SA65	53.62	<0.001
EFA vs SAG	-42.38	<0.001
EFA vs SCAP	21.62	<0.001
EFA vs SE	36.12	<0.001
EFA vs SE1	3.25	0.250
EFA vs SE2	-20.25	<0.001
EFA vs SH	13.62	<0.001
EFA vs SM	-50.38	<0.001
EFA vs SPY	-33.88	<0.001
EFA vs SSA	-24.50	<0.001
EFA vs SX	33.12	<0.001
SA25 vs SA65	10.00	<0.001
SA25 vs SAG	-86.00	<0.001
SA25 vs SCAP	-22.00	<0.001
SA25 vs SE	-7.50	0.002
SA25 vs SE1	-40.38	<0.001
SA25 vs SE2	-63.88	<0.001
SA25 vs SH	-30.00	<0.001
SA25 vs SM	-94.00	<0.001
SA25 vs SPY	-77.50	<0.001
SA25 vs SSA	-68.12	<0.001
SA25 vs SX	-10.50	<0.001
SA65 vs SAG	-96.00	<0.001
SA65 vs SCAP	-32.00	<0.001

Comparison	Estimate	Holm-adjusted p-value
SA65 vs SE	-17.50	<0.001
SA65 vs SE1	-50.38	<0.001
SA65 vs SE2	-73.88	<0.001
SA65 vs SH	-40.00	<0.001
SA65 vs SM	-104.00	<0.001
SA65 vs SPY	-87.50	<0.001
SA65 vs SSA	-78.12	<0.001
SA65 vs SX	-20.50	<0.001
SAG vs SCAP	64.00	<0.001
SAG vs SE	78.50	<0.001
SAG vs SE1	45.62	<0.001
SAG vs SE2	22.12	<0.001
SAG vs SH	56.00	<0.001
SAG vs SM	-8.00	0.002
SAG vs SPY	8.50	0.001
SAG vs SSA	17.88	<0.001
SAG vs SX	75.50	<0.001
SCAP vs SE	14.50	<0.001
SCAP vs SE1	-18.38	<0.001
SCAP vs SE2	-41.88	<0.001
SCAP vs SH	-8.00	0.002
SCAP vs SM	-72.00	<0.001
SCAP vs SPY	-55.50	<0.001
SCAP vs SSA	-46.12	<0.001
SCAP vs SX	11.50	<0.001
SE vs SE1	-32.88	<0.001
SE vs SE2	-56.38	<0.001
SE vs SH	-22.50	<0.001
SE vs SM	-86.50	<0.001
SE vs SPY	-70.00	<0.001
SE vs SSA	-60.62	<0.001
SE vs SX	-3.00	0.250
SE1 vs SE2	-23.50	<0.001
SE1 vs SH	10.38	<0.001
SE1 vs SM	-53.62	<0.001
SE1 vs SPY	-37.12	<0.001
SE1 vs SSA	-27.75	<0.001
SE1 vs SX	29.88	<0.001
SE2 vs SH	33.88	<0.001
SE2 vs SM	-30.12	<0.001
SE2 vs SPY	-13.62	<0.001
SE2 vs SSA	-4.25	0.137
SE2 vs SX	53.38	<0.001

Comparison	Estimate	Holm-adjusted p-value
SH vs SM	-64.00	<0.001
SH vs SPY	-47.50	<0.001
SH vs SSA	-38.12	<0.001
SH vs SX	19.50	<0.001
SM vs SPY	16.50	<0.001
SM vs SSA	25.88	<0.001
SM vs SX	83.50	<0.001
SPY vs SSA	9.38	0.0002
SPY vs SX	67.00	<0.001
SSA vs SX	57.62	<0.001

Table S7. Pairwise comparisons of cocci-shaped bacterial strains at 48 h based on mean hue values (ART post hoc test with Holm correction).

Comparison	Estimate	Holm-adjusted p-value
EF vs EFA	-8.875	0.054
EF vs SA25	-37.000	<0.001
EF vs SA65	29.875	<0.001
EF vs SAG	-52.000	<0.001
EF vs SCAP	12.000	0.003
EF vs SE	-10.375	0.016
EF vs SE1	34.125	<0.001
EF vs SE2	-34.750	<0.001
EF vs SH	-60.000	<0.001
EF vs SM	20.000	<0.001
EF vs SPY	-35.625	<0.001
EF vs SSA	-13.375	0.001
EF vs SX	44.000	<0.001
EFA vs SA25	-28.125	<0.001
EFA vs SA65	38.750	<0.001
EFA vs SAG	-43.125	<0.001
EFA vs SCAP	20.875	<0.001
EFA vs SE	-1.500	1.000
EFA vs SE1	43.000	<0.001
EFA vs SE2	-25.875	<0.001
EFA vs SH	-51.125	<0.001
EFA vs SM	28.875	<0.001
EFA vs SPY	-26.750	<0.001
EFA vs SSA	-4.500	1.000
EFA vs SX	52.875	<0.001
SA25 vs SA65	66.875	<0.001
SA25 vs SAG	-15.000	<0.001
SA25 vs SCAP	49.000	<0.001
SA25 vs SE	26.625	<0.001
SA25 vs SE1	71.125	<0.001

Comparison	Estimate	Holm-adjusted p-value
SA25 vs SE2	2.250	1.000
SA25 vs SH	-23.000	<0.001
SA25 vs SM	57.000	<0.001
SA25 vs SPY	1.375	1.000
SA25 vs SSA	23.625	<0.001
SA25 vs SX	81.000	<0.001
SA65 vs SAG	-81.875	<0.001
SA65 vs SCAP	-17.875	<0.001
SA65 vs SE	-40.250	<0.001
SA65 vs SE1	4.250	1.000
SA65 vs SE2	-64.625	<0.001
SA65 vs SH	-89.875	<0.001
SA65 vs SM	-9.875	0.025
SA65 vs SPY	-65.500	<0.001
SA65 vs SSA	-43.250	<0.001
SA65 vs SX	14.125	<0.001
SAG vs SCAP	64.000	<0.001
SAG vs SE	41.625	<0.001
SAG vs SE1	86.125	<0.001
SAG vs SE2	17.250	<0.001
SAG vs SH	-8.000	0.107
SAG vs SM	72.000	<0.001
SAG vs SPY	16.375	<0.001
SAG vs SSA	38.625	<0.001
SAG vs SX	96.000	<0.001
SCAP vs SE	-22.375	<0.001
SCAP vs SE1	22.125	<0.001
SCAP vs SE2	-46.750	<0.001
SCAP vs SH	-72.000	<0.001
SCAP vs SM	8.000	0.107
SCAP vs SPY	-47.625	<0.001
SCAP vs SSA	-25.375	<0.001
SCAP vs SX	32.000	<0.001
SE vs SE1	44.500	<0.001
SE vs SE2	-24.375	<0.001
SE vs SH	-49.625	<0.001
SE vs SM	30.375	<0.001
SE vs SPY	-25.250	<0.001
SE vs SSA	-3.000	1.000
SE vs SX	54.375	<0.001
SE1 vs SE2	-68.875	<0.001
SE1 vs SH	-94.125	<0.001
SE1 vs SM	-14.125	<0.001

Comparison	Estimate	Holm-adjusted p-value
SE1 vs SPY	-69.750	<0.001
SE1 vs SSA	-47.500	<0.001
SE1 vs SX	9.875	0.025
SE2 vs SH	-25.250	<0.001
SE2 vs SM	54.750	<0.001
SE2 vs SPY	-0.875	1.000
SE2 vs SSA	21.375	<0.001
SE2 vs SX	78.750	<0.001
SH vs SM	80.000	<0.001
SH vs SPY	24.375	<0.001
SH vs SSA	46.625	<0.001
SH vs SX	104.000	<0.001
SM vs SPY	-55.625	<0.001
SM vs SSA	-33.375	<0.001
SM vs SX	24.000	<0.001
SPY vs SSA	22.250	<0.001
SPY vs SX	79.625	<0.001
SSA vs SX	57.375	<0.001

Table S8. Pairwise comparisons of cocci-shaped bacterial strains at 72 h based on mean hue values (ART post hoc test with Holm correction).

Comparison	Estimate	Holm-adjusted p-value
EF vs EFA	24.750	<0.001
EF vs SA25	58.625	<0.001
EF vs SA65	47.000	<0.001
EF vs SAG	-16.625	<0.001
EF vs SCAP	47.875	<0.001
EF vs SE	-7.625	0.259
EF vs SE1	-25.375	<0.001
EF vs SE2	19.000	<0.001
EF vs SH	26.000	<0.001
EF vs SM	2.875	1.000
EF vs SPY	47.000	<0.001
EF vs SSA	-33.375	<0.001
EF vs SX	70.625	<0.001
EFA vs SA25	33.875	<0.001
EFA vs SA65	22.250	<0.001
EFA vs SAG	-41.375	<0.001
EFA vs SCAP	23.125	<0.001
EFA vs SE	-32.375	<0.001
EFA vs SE1	-50.125	<0.001
EFA vs SE2	-5.750	0.630

EFA vs SH	1.250	1.000
EFA vs SM	-21.875	<0.001
EFA vs SPY	22.250	<0.001
EFA vs SSA	-58.125	<0.001
EFA vs SX	45.875	<0.001
SA25 vs SA65	-11.625	0.020
SA25 vs SAG	-75.250	<0.001
SA25 vs SCAP	-10.750	0.037
SA25 vs SE	-66.250	<0.001
SA25 vs SE1	-84.000	<0.001
SA25 vs SE2	-39.625	<0.001
SA25 vs SH	-32.625	<0.001
SA25 vs SM	-55.750	<0.001
SA25 vs SPY	-11.625	0.020
SA25 vs SSA	-92.000	<0.001
SA25 vs SX	12.000	0.015
SA65 vs SAG	-63.625	<0.001
SA65 vs SCAP	0.875	1.000
SA65 vs SE	-54.625	<0.001
SA65 vs SE1	-72.375	<0.001
SA65 vs SE2	-28.000	<0.001
SA65 vs SH	-21.000	<0.001
SA65 vs SM	-44.125	<0.001
SA65 vs SPY	0.000	1.000
SA65 vs SSA	-80.375	<0.001
SA65 vs SX	23.625	<0.001
SAG vs SCAP	64.500	<0.001
SAG vs SE	9.000	0.132
SAG vs SE1	-8.750	0.145
SAG vs SE2	35.625	<0.001
SAG vs SH	42.625	<0.001
SAG vs SM	19.500	<0.001
SAG vs SPY	63.625	<0.001
SAG vs SSA	-16.750	<0.001
SAG vs SX	87.250	<0.001
SCAP vs SE	-55.500	<0.001
SCAP vs SE1	-73.250	<0.001
SCAP vs SE2	-28.875	<0.001
SCAP vs SH	-21.875	<0.001
SCAP vs SM	-45.000	<0.001
SCAP vs SPY	-0.875	1.000
SCAP vs SSA	-81.250	<0.001
SCAP vs SX	22.750	<0.001
SE vs SE1	-17.750	<0.001

SE vs SE2	26.625	<0.001
SE vs SH	33.625	<0.001
SE vs SM	10.500	0.043
SE vs SPY	54.625	<0.001
SE vs SSA	-25.750	<0.001
SE vs SX	78.250	<0.001
SE1 vs SE2	44.375	<0.001
SE1 vs SH	51.375	<0.001
SE1 vs SM	28.250	<0.001
SE1 vs SPY	72.375	<0.001
SE1 vs SSA	-8.000	0.225
SE1 vs SX	96.000	<0.001
SE2 vs SH	7.000	0.344
SE2 vs SM	-16.125	0.0002
SE2 vs SPY	28.000	<0.001
SE2 vs SSA	-52.375	<0.001
SE2 vs SX	51.625	<0.001
SH vs SM	-23.125	<0.001
SH vs SPY	21.000	<0.001
SH vs SSA	-59.375	<0.001
SH vs SX	44.625	<0.001
SM vs SPY	44.125	<0.001
SM vs SSA	-36.250	<0.001
SM vs SX	67.750	<0.001
SPY vs SSA	-80.375	<0.001
SPY vs SX	23.625	<0.001
SSA vs SX	104.000	<0.001

Table S9. Pairwise comparisons of cocci-shaped bacterial strains at 96 h based on mean hue values (ART post hoc test with Holm correction).

Comparison	Estimate	Holm-adjusted p-value
EF vs EFA	-25.000	<0.001
EF vs SA25	-35.750	<0.001
EF vs SA65	22.750	<0.001
EF vs SAG	30.250	<0.001
EF vs SCAP	9.375	0.027
EF vs SE	-38.625	<0.001
EF vs SE1	-64.375	<0.001
EF vs SE2	-57.625	<0.001
EF vs SH	-48.625	<0.001
EF vs SM	-17.000	<0.001
EF vs SPY	39.000	<0.001
EF vs SSA	1.500	1.000

EF vs SX	2.125	1.000
EFA vs SA25	-10.750	0.007
EFA vs SA65	47.750	<0.001
EFA vs SAG	55.250	<0.001
EFA vs SCAP	34.375	<0.001
EFA vs SE	-13.625	<0.001
EFA vs SE1	-39.375	<0.001
EFA vs SE2	-32.625	<0.001
EFA vs SH	-23.625	<0.001
EFA vs SM	8.000	0.079
EFA vs SPY	64.000	<0.001
EFA vs SSA	26.500	<0.001
EFA vs SX	27.125	<0.001
SA25 vs SA65	58.500	<0.001
SA25 vs SAG	66.000	<0.001
SA25 vs SCAP	45.125	<0.001
SA25 vs SE	-2.875	1.000
SA25 vs SE1	-28.625	<0.001
SA25 vs SE2	-21.875	<0.001
SA25 vs SH	-12.875	<0.001
SA25 vs SM	18.750	<0.001
SA25 vs SPY	74.750	<0.001
SA25 vs SSA	37.250	<0.001
SA25 vs SX	37.875	<0.001
SA65 vs SAG	7.500	0.096
SA65 vs SCAP	-13.375	<0.001
SA65 vs SE	-61.375	<0.001
SA65 vs SE1	-87.125	<0.001
SA65 vs SE2	-80.375	<0.001
SA65 vs SH	-71.375	<0.001
SA65 vs SM	-39.750	<0.001
SA65 vs SPY	16.250	<0.001
SA65 vs SSA	-21.250	<0.001
SA65 vs SX	-20.625	<0.001
SAG vs SCAP	-20.875	<0.001
SAG vs SE	-68.875	<0.001
SAG vs SE1	-94.625	<0.001
SAG vs SE2	-87.875	<0.001
SAG vs SH	-78.875	<0.001
SAG vs SM	-47.250	<0.001
SAG vs SPY	8.750	0.043
SAG vs SSA	-28.750	<0.001
SAG vs SX	-28.125	<0.001
SCAP vs SE	-48.000	<0.001

SCAP vs SE1	-73.750	<0.001
SCAP vs SE2	-67.000	<0.001
SCAP vs SH	-58.000	<0.001
SCAP vs SM	-26.375	<0.001
SCAP vs SPY	29.625	<0.001
SCAP vs SSA	-7.875	0.079
SCAP vs SX	-7.250	0.103
SE vs SE1	-25.750	<0.001
SE vs SE2	-19.000	<0.001
SE vs SH	-10.000	0.015
SE vs SM	21.625	<0.001
SE vs SPY	77.625	<0.001
SE vs SSA	40.125	<0.001
SE vs SX	40.750	<0.001
SE1 vs SE2	6.750	0.131
SE1 vs SH	15.750	<0.001
SE1 vs SM	47.375	<0.001
SE1 vs SPY	103.375	<0.001
SE1 vs SSA	65.875	<0.001
SE1 vs SX	66.500	<0.001
SE2 vs SH	9.000	0.037
SE2 vs SM	40.625	<0.001
SE2 vs SPY	96.625	<0.001
SE2 vs SSA	59.125	<0.001
SE2 vs SX	59.750	<0.001
SH vs SM	31.625	<0.001
SH vs SPY	87.625	<0.001
SH vs SSA	50.125	<0.001
SH vs SX	50.750	<0.001
SM vs SPY	56.000	<0.001
SM vs SSA	18.500	<0.001
SM vs SX	19.125	<0.001
SPY vs SSA	-37.500	<0.001
SPY vs SX	-36.875	<0.001
SSA vs SX	0.625	1.000

Table S10. Pairwise comparisons of cocci-shaped bacterial strains at 168 h based on mean hue values (ART post hoc test with Holm correction).

Comparison	Estimate	Holm-adjusted p-value
EF vs EFA	6.50	0.258
EF vs SA25	-21.62	<0.001
EF vs SA65	-53.88	<0.001
EF vs SAG	42.12	<0.001
EF vs SCAP	-9.25	0.091

Comparison	Estimate	Holm-adjusted p-value
EF vs SE	50.12	<0.001
EF vs SE1	18.38	<0.001
EF vs SE2	-32.50	<0.001
EF vs SH	-0.75	0.862
EF vs SM	-45.88	<0.001
EF vs SPY	25.62	<0.001
EF vs SSA	30.12	<0.001
EF vs SX	-35.25	<0.001
EFA vs SA25	-28.12	<0.001
EFA vs SA65	-60.38	<0.001
EFA vs SAG	35.62	<0.001
EFA vs SCAP	-15.75	0.0003
EFA vs SE	43.62	<0.001
EFA vs SE1	11.88	0.013
EFA vs SE2	-39.00	<0.001
EFA vs SH	-7.25	0.238
EFA vs SM	-52.38	<0.001
EFA vs SPY	19.12	<0.001
EFA vs SSA	23.62	<0.001
EFA vs SX	-41.75	<0.001
SA25 vs SA65	-32.25	<0.001
SA25 vs SAG	63.75	<0.001
SA25 vs SCAP	12.38	0.009
SA25 vs SE	71.75	<0.001
SA25 vs SE1	40.00	<0.001
SA25 vs SE2	-10.88	0.028
SA25 vs SH	20.88	<0.001
SA25 vs SM	-24.25	<0.001
SA25 vs SPY	47.25	<0.001
SA25 vs SSA	51.75	<0.001
SA25 vs SX	-13.62	0.003
SA65 vs SAG	96.00	<0.001
SA65 vs SCAP	44.62	<0.001
SA65 vs SE	104.00	<0.001
SA65 vs SE1	72.25	<0.001
SA65 vs SE2	21.38	<0.001
SA65 vs SH	53.12	<0.001
SA65 vs SM	8.00	0.188
SA65 vs SPY	79.50	<0.001
SA65 vs SSA	84.00	<0.001
SA65 vs SX	18.62	<0.001
SAG vs SCAP	-51.38	<0.001
SAG vs SE	8.00	0.188

Comparison	Estimate	Holm-adjusted p-value
SAG vs SE1	-23.75	<0.001
SAG vs SE2	-74.62	<0.001
SAG vs SH	-42.88	<0.001
SAG vs SM	-88.00	<0.001
SAG vs SPY	-16.50	0.0001
SAG vs SSA	-12.00	0.012
SAG vs SX	-77.38	<0.001
SCAP vs SE	59.38	<0.001
SCAP vs SE1	27.62	<0.001
SCAP vs SE2	-23.25	<0.001
SCAP vs SH	8.50	0.146
SCAP vs SM	-36.62	<0.001
SCAP vs SPY	34.88	<0.001
SCAP vs SSA	39.38	<0.001
SCAP vs SX	-26.00	<0.001
SE vs SE1	-31.75	<0.001
SE vs SE2	-82.62	<0.001
SE vs SH	-50.88	<0.001
SE vs SM	-96.00	<0.001
SE vs SPY	-24.50	<0.001
SE vs SSA	-20.00	<0.001
SE vs SX	-85.38	<0.001
SE1 vs SE2	-50.88	<0.001
SE1 vs SH	-19.12	<0.001
SE1 vs SM	-64.25	<0.001
SE1 vs SPY	7.25	0.238
SE1 vs SSA	11.75	0.014
SE1 vs SX	-53.62	<0.001
SE2 vs SH	31.75	<0.001
SE2 vs SM	-13.38	0.004
SE2 vs SPY	58.12	<0.001
SE2 vs SSA	62.62	<0.001
SE2 vs SX	-2.75	0.862
SH vs SM	-45.12	<0.001
SH vs SPY	26.38	<0.001
SH vs SSA	30.88	<0.001
SH vs SX	-34.50	<0.001
SM vs SPY	71.50	<0.001
SM vs SSA	76.00	<0.001
SM vs SX	10.62	0.032
SPY vs SSA	4.50	0.596
SPY vs SX	-60.88	<0.001
SSA vs SX	-65.38	<0.001

Comparison	Estimate	Holm-adjusted p-value

Table S11. Statistical workflow and R functions applied in the analysis of bacterial hue values.

Analysis step	Statistical method / R function
Data import	readxl::read_excel()
Conversion of categorical variables	factor()
Assessment of normality	Shapiro–Wilk test (shapiro.test)
Assessment of homogeneity of variance	Levene's test (car::leveneTest)
Outlier inspection	Boxplots (1.5×IQR criterion)
Main analysis for H1–H3	ART ANOVA (ARTool::art)
ANOVA table generation	anova()
Post hoc comparisons between strains	ART contrasts (art.con)
Multiple comparison correction	Holm correction
Data visualization	ggplot2
Planned contrasts for H4	ART contrasts (art.con)
Gram-positive vs Gram-negative comparison	art(ΔH ~ Gram)
Cocci vs rods comparison	art(ΔH ~ Typ)

Figure S1. Boxplots used for visual assessment of outliers in bacilli strains

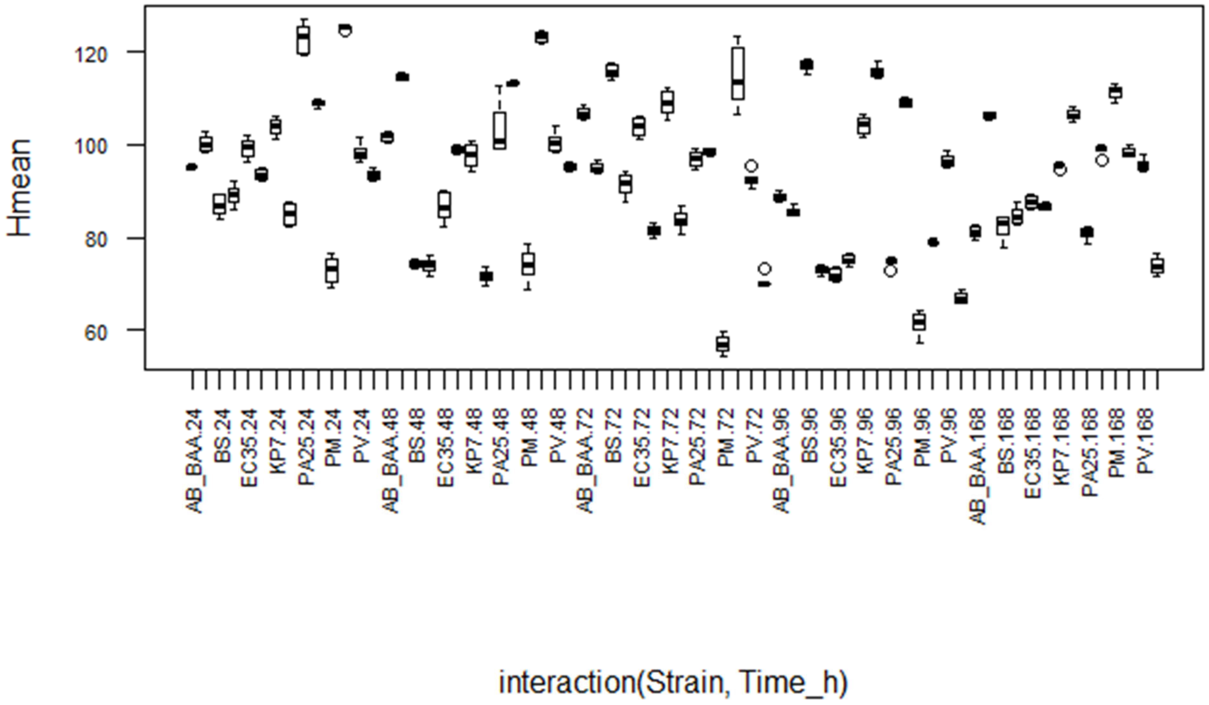

Boxplots of mean hue values (Hmean) for bacilli strains across incubation times used for visual assessment of potential outliers prior to ART ANOVA analysis. Outliers were assessed according to the 1.5×IQR criterion. No extreme outliers requiring exclusion from the analysis were identified.

Figure S2. Boxplots used for visual assessment of outliers in coccal bacterial strains.

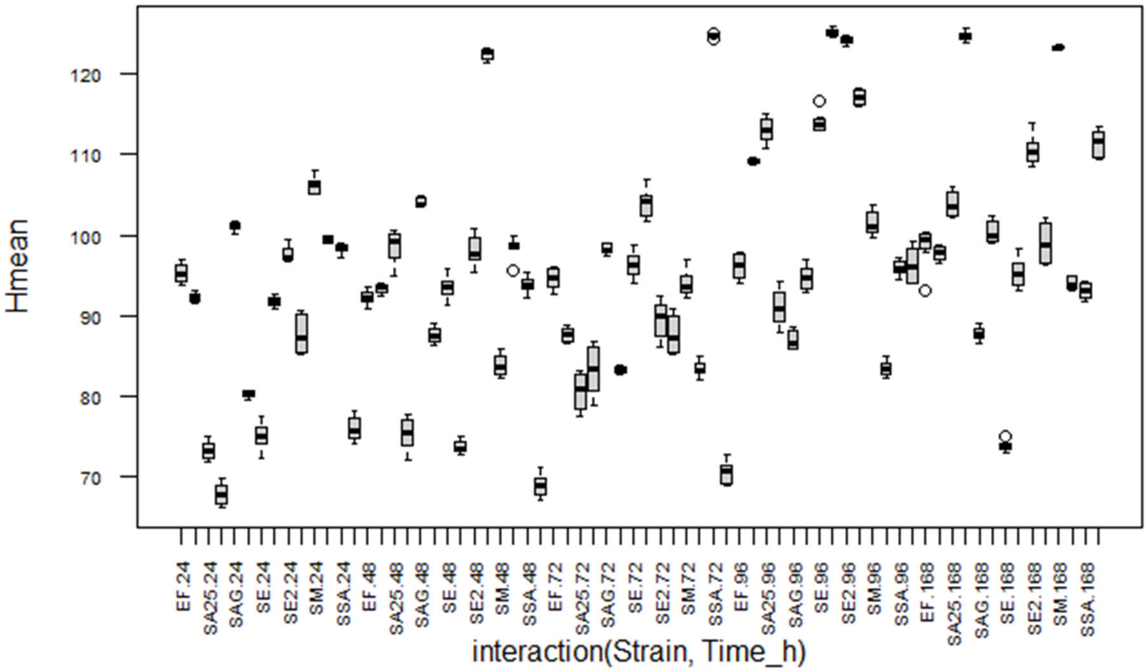

Boxplots of mean hue values (Hmean) for bacilli strains across incubation times used for visual assessment of potential outliers prior to ART ANOVA analysis. Outliers were assessed according to the 1.5×IQR criterion. No extreme outliers requiring exclusion from the analysis were identified.
